# Supplementary material for: Receptor Activator of NF-κB Orchestrates Activation of Antiviral Memory CD8 T Cells in the Spleen Marginal Zone
Source: Cell Rep. 2017 Nov 28;21(9):2515–27. doi: 10.1016/j.celrep.2017.10.111 (PMC5723674; doi:10.1016/j.celrep.2017.10.111)
Supplement: Document S1. Supplemental Experimental Procedures and Figures S1–S6 [file mmc1.pdf]

Cell Reports, Volume *21*

Supplemental Information

Receptor Activator of NF-κB Orchestrates Activation of Antiviral Memory CD8 T Cells in the Spleen Marginal Zone

Mohamed Habbeddine, Christophe Verthuy, Olivia Rastoin, Lionel Chasson, Magali Bebien, Marc Bajenoff, Sahil Adriouch, Joke M.M. den Haan, Josef M. Penninger, and Toby Lawrence

Supplementary material

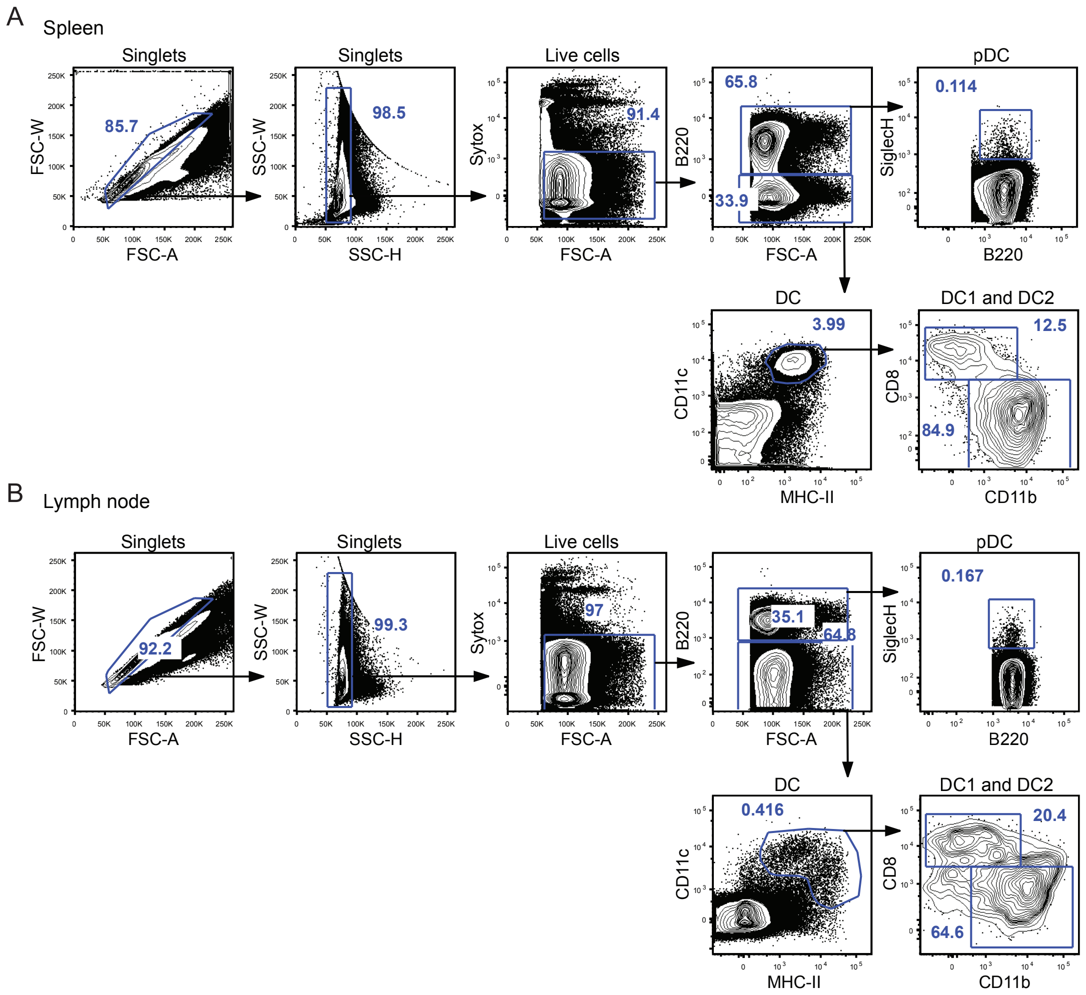

Figure S1. Detailed gating strategy for DC subsets, related to Figure 1. (A) gating strategy for DC1 (CD8α^{+}), DC2 (CD11b^{+}) and pDC (SiglecH^{+}) subsets in spleen (A) and lymph node (B).

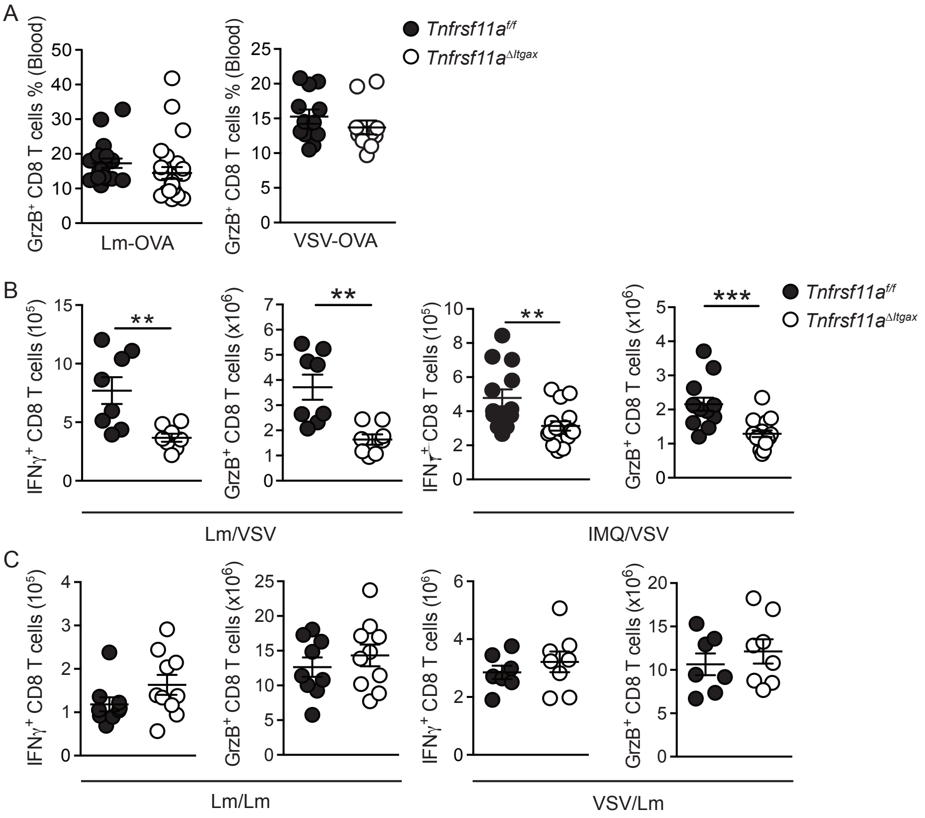

Figure S2. RANK expression by $CD11c^+$ cells regulates memory CD8 T cell activation in response to viral infection, related to Figure 2. (A) *Tnfrsf11a*$^{f/f}$ and *Tnfrsf11a*$^{\Delta Itgax}$ mice were infected i.v. with $1x10^4$ Cfu Lm-OVA or $1x10^5$ Pfu VSV-OVA; 1 week later blood was collected and granzyme B (GrzB) production by was measured in $CD8^+$ T cells by intracellular cytokine staining (ICS), after *ex vivo* stimulation with cognate peptide (SIINFEKL). (B-C) Cohorts of *Tnfrsf11a*$^{f/f}$ and *Tnfrsf11a*$^{\Delta Itgax}$ mice were immunized with Lm-OVA, IMQ/OVA or VSV-OVA; 2 months later mice were challenged with either VSV-OVA (B) or Lm-OVA (C), 5 days later, IFNγ and GrzB production were measured in spleen $CD8^+$ T cells by ICS, after *ex vivo* stimulation with SIINFEKL. Data are represented as mean ± s.e.m. and statistical analysis was performed with Mann–Whitney test; **p=<0.01, ***p=<0.005.

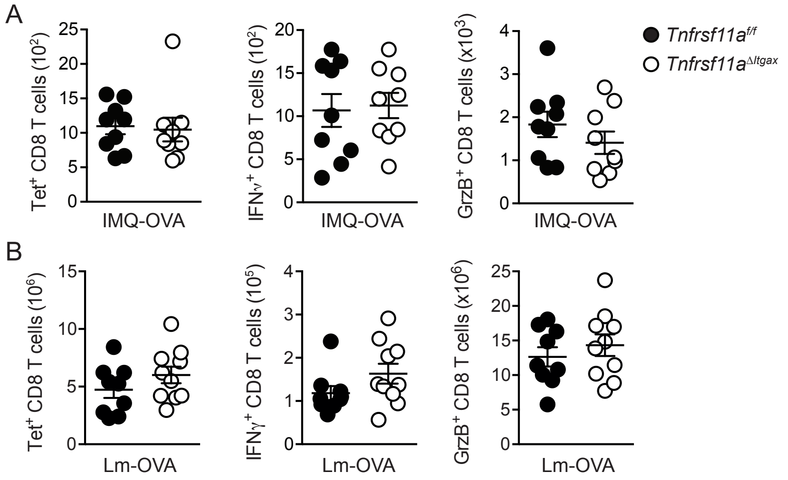

Figure S3. RANK in CD11c^{+} cells is not required for the development of pathogen-specific mCTL, related to Figure 2. (A-B) Cohorts of *Tnfrsf11a$^{f/f}$* and *Tnfrsf11a$^{\Delta Itgax}$* mice were immunized with IMQ/OVA (A) or Lm-OVA (B); 2 months later spleen cells were analysed by FACS for expansion of OVA-specific memory CD8 T cells (CD44^{+} Tet^{+}) and IFNγ or GrzB expressing cells upon activation with SIINFEKL. Data are represented as mean ± s.e.m.

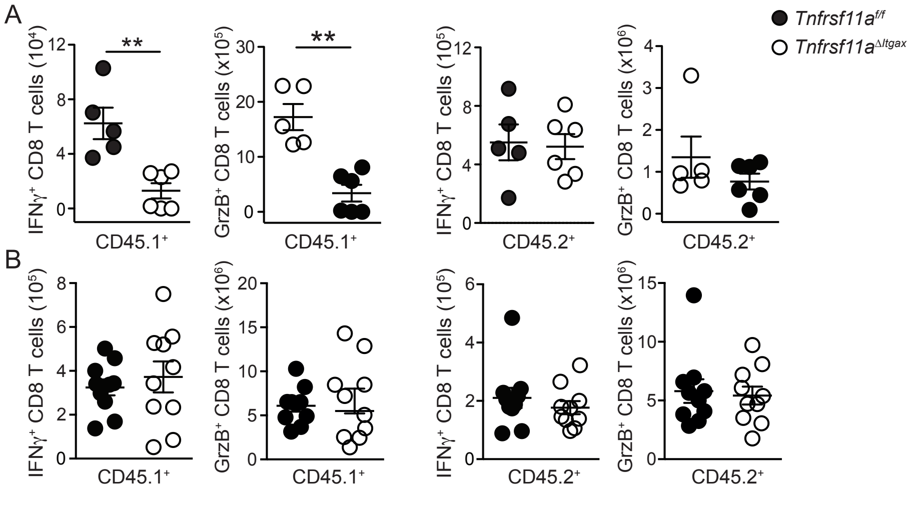

Figure S4. RANK in CD11c^{+} cells regulates activation of adoptively transferred memory CD8 T cells, related to Figure 2. (A-B) CD45.1^{+} mice were immunized with Lm-OVA, 3 weeks later CD4 and CD8 T cells were isolated from spleen and adoptively transferred to cohorts of naïve *Tnfrsf11a$^{f/f}$* and *Tnfrsf11a$^{\Delta Itgax}$* mice, a further 7 weeks after T cell transfer mice were infected with VSV-OVA (A) or Lm-OVA (B); IFNγ and GrzB expression in adoptively transferred mCTL (CD44^{+}, CD45.1^{+}) and endogenous naïve CD8 T cells (CD45.2^{+}) cells was measured 5 days after challenge in spleen, after stimulation with cognate peptide (SIINFEKL). Data are represented as mean ± s.e.m. and statistical analysis was performed with Mann–Whitney test; **p=<0.01.

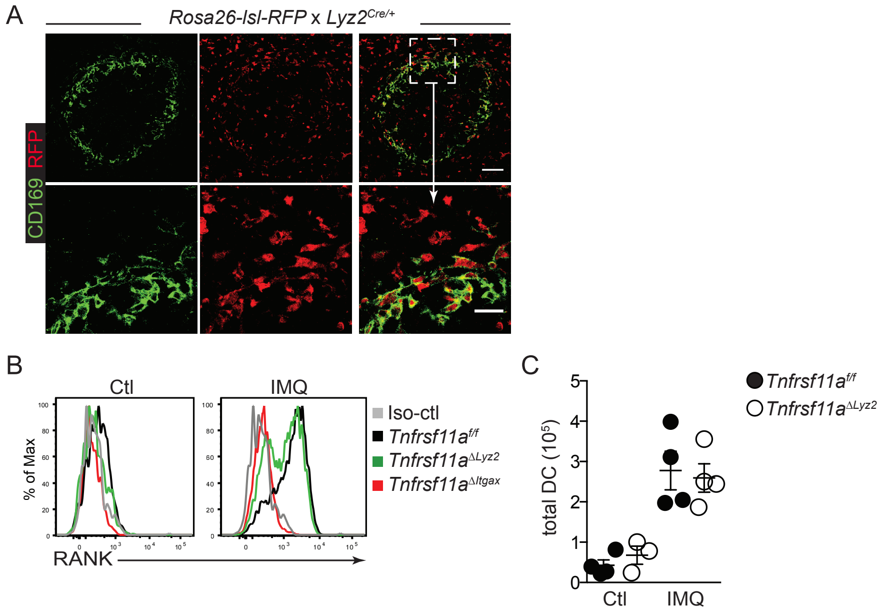

Figure S5. RANK expression in DC from *Tnfrsf11a*$^{\Delta Lyz2}$ mice, related to Figure 5. (A) Spleen sections from *Rosa26-LSL-tdRFP* x *Lyz2*$^{cre/+}$ mice showing Cre-recombinase activity in CD169^{+} cells; tdRFP (red) and CD169 (green). Scale bars; 50 µm in upper panels and 25 µm in lower panels. (B) RANK expression on DC (MHC-II^{hi} CD11c^{+}) was measured by flow cytometry in CLN from *Tnfrsf11a*$^{f/f}$, *Tnfrsf11a*$^{\Delta Lyz2}$ and *Tnfrsf11a*$^{\Delta Itgax}$ mice with and without IMQ treatment, representative FACs plots are shown. (B) Total number of DC were quantified. Representative data from at least 2 independent experiments are shown.

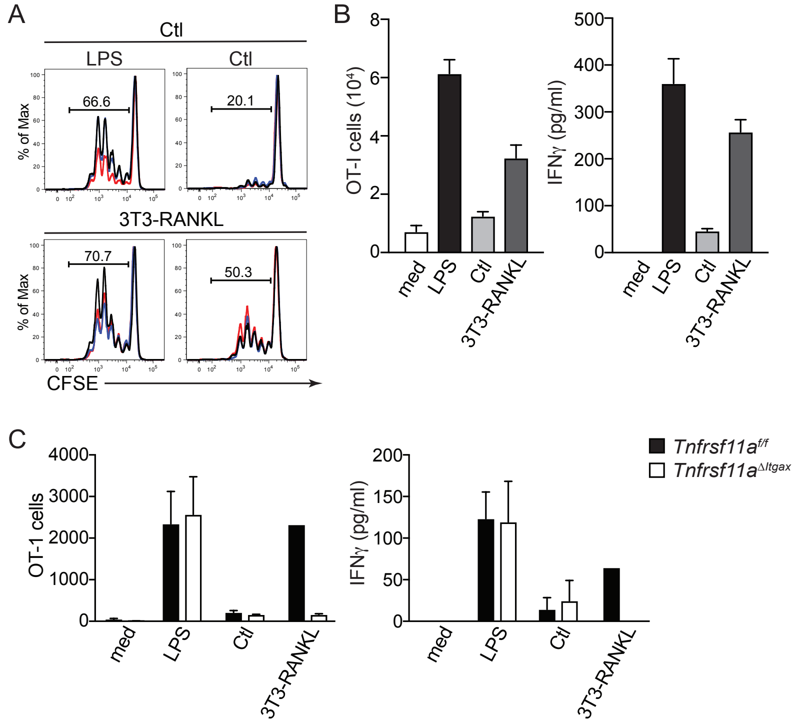

Figure S6. RANKL increases cross-presenting activity by DC *in vitro*, related to Figure 6. (A-B) Bone marrow-derived DC (BMDC) for wild-type mice (A-B), or *Tnfrsf11a*$^{f/f}$ and *Tnfrsf11a*$^{\Delta Itgax}$ mice (C), were co-cultured with mouse fibroblasts expressing RANKL (3T3-RANKL), control cells (Ctl) or 100ng/ml LPS, before loading with recombinant OVA and addition of CFSE labeled OVA-specific T cells (OT-1). After 3 days, CFSE dilution was analysed by flow cytometry and the number of proliferating OT-1 cells quantified. IFNγ production

by OT-1 cells was measured by ELISA. Data are represented as mean ± s.e.m. of cumulative data from 3 independent experiments.

Supplementary experimental procedures.

Generation of RANKL expressing stromal cells and cross-presentation assays.
3T3-RANKL cell line was generated as described by Sarrazin et al. (Sarrazin et al., 2009). Briefly, MSCV-based retroviral vectors were constructed by PCR cloning of cDNA for mouse RANKL under LTR control and by replacement of the IRES-driven GFP to generate RANKL-GFP. Viral supernatants was produced with ϕNXe cells (http://www.stanford.edu/group/nolan) and used to infect 3T3 cells by a double spin infection for 2 h in the presence of 8 mg/ml polybrene. Bone marrow-derived dendritic cells (BMDC) were generated according to the Steinman protocol (Inaba et al., 1992) . Briefly, cells were cultured with 20 ng/ml recombinant mouse GM-CSF (Peprotech) the medium was changed at day 2 and 4 with fresh medium supplemented with GM-CSF. BMDC were collected at day 6 and co-cultured overnight on top of 3T3-RANKL or Ctl cells at a ratio 45:1. Non-adherent DC were harvested and washed before incubation with recombinant OVA in culture medium for 1-2 hours. DC were then washed extensively with culture medium before addition of OT-1 T cells labelled with CFSE at a ratio 1:3 for 2.5 days, CFSE dilution as an index of T cell proliferation was analysed by flow cytometry.

Inaba, K., Inaba, M., Romani, N., Aya, H., Deguchi, M., Ikehara, S., Muramatsu, S., and Steinman, R.M. (1992). Generation of large numbers of dendritic cells from mouse bone marrow cultures supplemented with granulocyte/macrophage colony-stimulating factor. The Journal of experimental medicine *176*, 1693-1702.

Sarrazin, S., Mossadegh-Keller, N., Fukao, T., Aziz, A., Mourcin, F., Vanhille, L., Kelly Modis, L., Kastner, P., Chan, S., Duprez, E., *et al.* (2009). MafB restricts M-CSF-dependent myeloid commitment divisions of hematopoietic stem cells. Cell *138*, 300-313.
