# Supplementary material for: Receptor Activator of NF-κB Orchestrates Activation of Antiviral Memory CD8 T Cells in the Spleen Marginal Zone
Source: Cell Rep. 2017 Nov 28;21(9):2515–27. doi: 10.1016/j.celrep.2017.10.111 (PMC5723674; doi:10.1016/j.celrep.2017.10.111)
Supplement: Document S2. Article plus Supplemental Information [file mmc2.pdf]

Cell Reports

Article

Receptor Activator of NF-κB Orchestrates Activation of Antiviral Memory CD8 T Cells in the Spleen Marginal Zone

Graphical Abstract

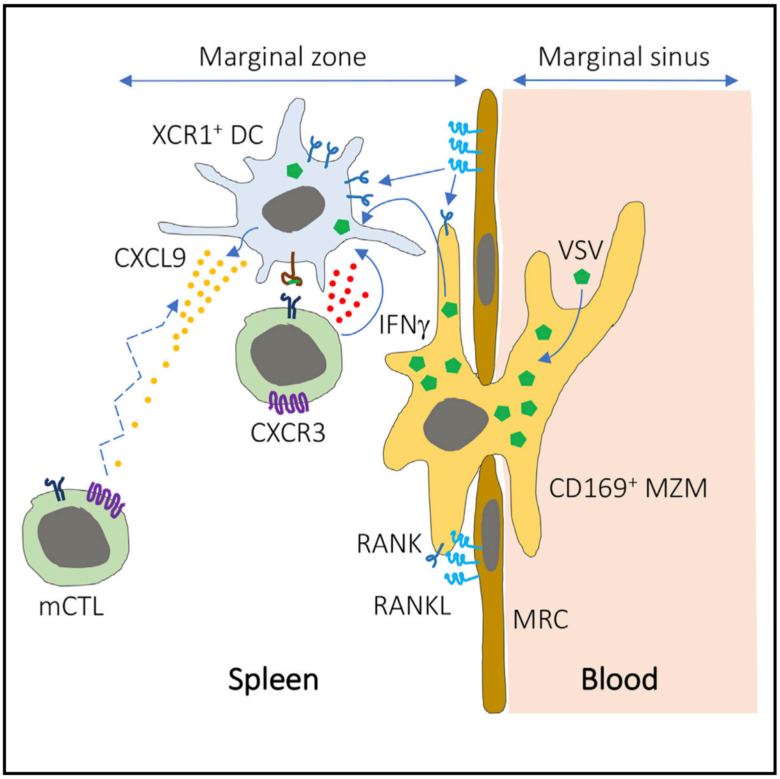

Highlights

- RANK deletion does not affect DC survival or T cell priming in the context of infection
- RANK is required for memory CD8 T cell activation in response to viral infection
- RANK-dependent CD169$^+$ macrophages support viral replication in the spleen marginal zone
- RANK expression in DCs promotes the recruitment and activation of memory CD8 T cells

Authors

Mohamed Habbeddine, Christophe Verthuy, Olivia Rastoin, ..., Joke M.M. den Haan, Josef M. Penninger, Toby Lawrence

Correspondence

lawrence@ciml.univ-mrs.fr

In Brief

Habbeddine et al. show that RANK signaling in the spleen marginal zone plays an important role in orchestrating protective immune responses to viral infection. They show that RANK expressed by macrophages supports viral replication, while RANK signaling in dendritic cells promotes the recruitment and activation of memory CD8 T cells.

Habbeddine et al., 2017, Cell Reports 21, 2515–2527
November 28, 2017 © 2017 The Author(s).
https://doi.org/10.1016/j.celrep.2017.10.111

OPEN ACCESS
CellPress

Receptor Activator of NF-κB Orchestrates Activation of Antiviral Memory CD8 T Cells in the Spleen Marginal Zone

Mohamed Habbeddine,[1] Christophe Verthuy,[1] Olivia Rastoin,[1] Lionel Chasson,[1] Magali Bebien,[1] Marc Bajenoff,[1] Sahil Adriouch,[2,3] Joke M.M. den Haan,[4] Josef M. Penninger,[5] and Toby Lawrence[1,6,*]
[1]Centre d'Immunologie de Marseille-Luminy, Aix Marseille Université, Inserm, CNRS, Marseille, France
[2]Institute for Research and Innovation in Biomedicine (IRIB), University of Rouen, 76183 Rouen, France
[3]Institut National de la Santé et de la Recherche Médicale (Inserm), U905, 76183 Rouen, France
[4]Department of Molecular Cell Biology and Immunology, VU University Medical Center, 1007 MB Amsterdam, the Netherlands
[5]Institute of Molecular Biotechnology (IMBC), Austrian Academy of Sciences, 1030 Vienna, Austria
[6]Lead Contact
*Correspondence: lawrence@ciml.univ-mrs.fr
https://doi.org/10.1016/j.celrep.2017.10.111

SUMMARY

The spleen plays an important role in protective immunity to bloodborne pathogens. Macrophages and dendritic cells (DCs) in the spleen marginal zone capture microbial antigens to trigger adaptive immune responses. Marginal zone macrophages (MZMs) can also act as a replicative niche for intracellular pathogens, providing a platform for mounting the immune response. Here, we describe a role for RANK in the coordinated function of antigen-presenting cells in the spleen marginal zone and triggering anti-viral immunity. Targeted deletion of RANK results in the selective loss of CD169$^+$ MZMs, which provide a niche for viral replication, while RANK signaling in DCs promotes the recruitment and activation of anti-viral memory CD8 T cells. These studies reveal a role for the RANKL/RANK signaling axis in the orchestration of protective immune responses in the spleen marginal zone that has important implications for the host response to viral infection and induction of acquired immunity.

INTRODUCTION

Activation of memory CD8 T cells (mCTLs) is critical for protective immunity against intracellular pathogens such as viruses. Although memory T cells have an intrinsic capacity to respond rapidly upon secondary infection (Zhang and Bevan, 2011), triggering of memory cells in secondary lymphoid organs still requires the activity of professional antigen-presenting cells such as dendritic cells (DCs) (Alexandre et al., 2016; Zammit et al., 2005). mCTLs also occupy frontline niches, such as the spleen marginal zone (MZ), whereas naive T cells are confined to the follicular T cell zone (Schenkel et al., 2014). The spleen MZ is populated by antigen-presenting cells, including macrophages and DCs, that are strategically placed to capture pathogens and antigens from the blood (Martinez-Pomares and Gordon, 2012). Macrophages at the inner MZ, in direct contact with the marginal sinus and blood flow, are marked by expression of the lectin-like adhesion molecule Siglec-1 (CD169) and have been shown to play an important role in trapping immune complexes and antigen transfer to B cells (Martinez-Pomares and Gordon, 2012). Previous studies have shown that CD169-expressing cells can also promote the activation of CD8 T cells in certain contexts, implying a potential role in cell-mediated immunity. The specific ablation of CD169-expressing cells was shown to prevent cross-presentation of cell-associated viral and tumor antigens to CD8 T cells (Asano et al., 2011; Bernhard et al., 2015). Other studies have shown that antigen specifically targeted to CD169$^+$ cells can be transferred to CD8α-type DCs (DC1) in the spleen for cross-presentation to CD8 T cells (Backer et al., 2010). In the context of infection, CD169$^+$ MZ macrophages (MZMs) can be permissive for certain intracellular pathogens and form a restricted niche for replication, providing a platform for mounting both cell-mediated and humoral immune responses (Honke et al., 2011). In fact, CD169 itself has been shown to be a receptor for virus entry through binding to gangliosides in the viral envelope (Hammonds et al., 2017; Sewald et al., 2015). These studies suggest that CD169$^+$ MZMs, as well as DCs, can have important roles in CD8 T cell responses and cell-mediated immunity to bloodborne pathogens. However, the molecular mechanisms that regulate the coordinated function of these antigen-presenting cells and help orchestrate the protective immune response remain poorly understood.

Tumor necrosis factor (TNF) family cytokines lymphotoxin (LT), RANKL (TRANCE; TNFSF11), and TNF-α have important roles in the development of secondary lymphoid organs. Mice deficient in LT or RANKL signaling lack lymph nodes and have severe defects in the spleen microarchitecture (Weih and Caamaño, 2003). These defects are due to the loss of LT- and RANKL-mediated signaling cross-talk between hematopoietic lymphoid tissue inducer cells (LTis) and stromal lymphoid tissue organizer cells (LTos) during lymph node (LN) and spleen development (Brendolan and Caamaño, 2012; White et al., 2007). In adult mice, RANKL remains constitutively expressed by marginal reticular stromal cells (MRCs) (Jarjour et al., 2014; Katakai et al., 2008), which could be considered the adult equivalent of LTos. MRCs

CrossMark

This is an open access article under the CC BY license (http://creativecommons.org/licenses/by/4.0/).

OPEN ACCESS
CellPress

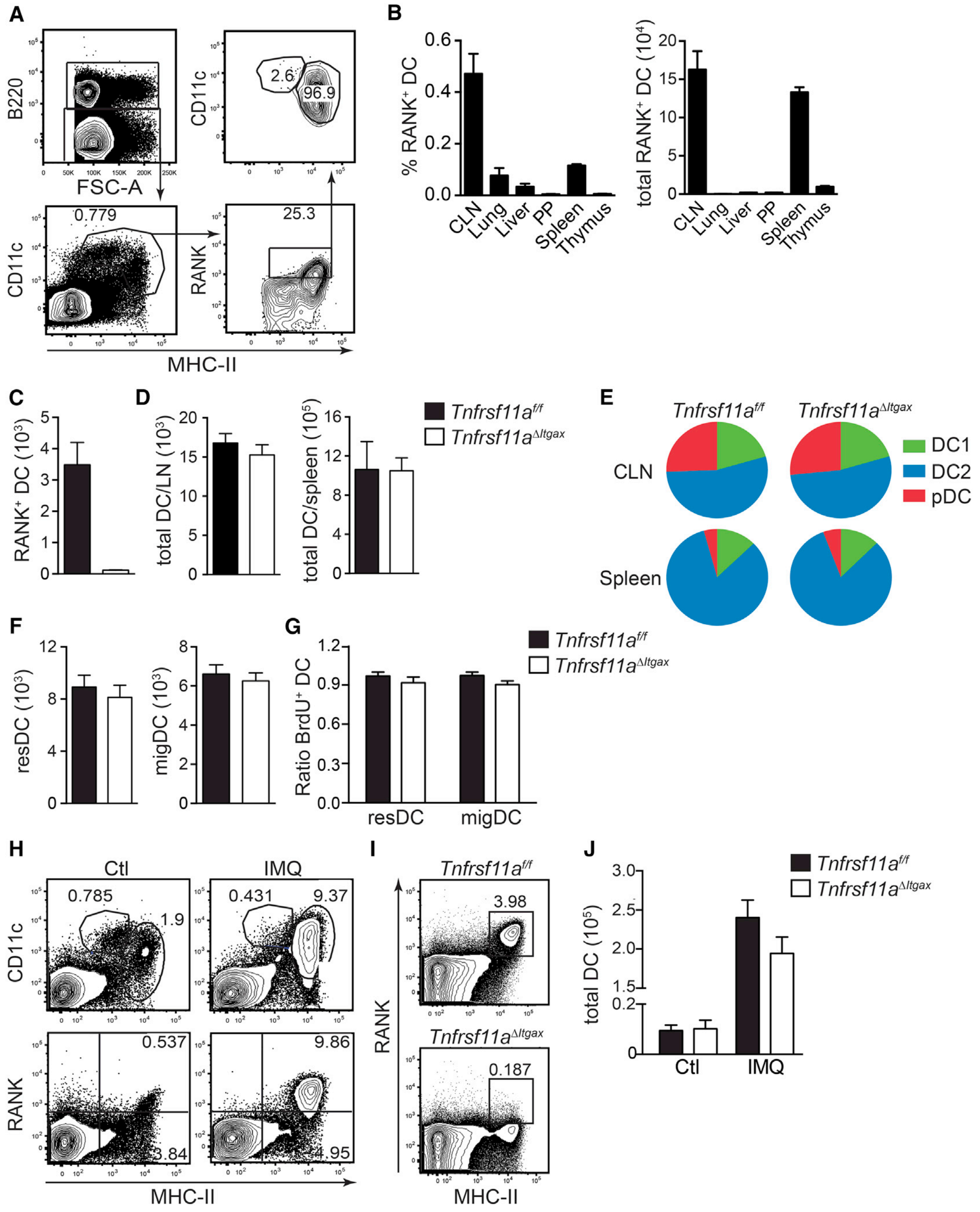

Figure 1. RANK Expression Does Not Affect DC Survival or Longevity

(A) Gating strategy for RANK$^+$ DCs; representative fluorescence-activated cell sorting (FACS) plots are shown.

(B) Proportion of RANK$^+$ DCs (MHC-II^{hi} CD11c$^+$ cells) among immune cells in different organs (CLN, cutaneous LN; MLN, mesenteric LN; PP, Peyer's patches).

(C) Number of RANK$^+$ DCs in CLN from *Tnfrsf11a$^{f/f}$* and *Tnfrsf11a$^{\Delta Itgax}$* mice.

(D) Total numbers of DCs in LN and spleen.

(E) Proportions of DC1, DC2, and pDC subsets in CLN and spleen.

(F) Relative numbers of resident DCs (resDC) and migratory DCs (migDC) in CLN.

(G) Lethally irradiated CD45.1$^+$ mice were reconstituted with a 1:1 mix of bone marrow cells from either *Tnfrsf11a$^{f/f}$* or *Tnfrsf11a$^{\Delta Itgax}$* mice (donor; CD45.2$^+$) and wild-type cells from CD45.2$^+$/CD45.1$^+$ mice (competitor). BrdU incorporation by MHC-II^{hi} CD11c$^+$ cells was measured in CLN from chimeric mice by flow cytometry; the ratio of BrdU$^+$ resDCs and migDCs from donor (*Tnfrsf11a$^{f/f}$* and *Tnfrsf11a$^{\Delta Itgax}$*) and competitor cells is shown.

(legend continued on next page)

line the marginal sinuses of the spleen and LNs juxtaposed with CD169$^+$ macrophages, but their function remains unclear. LT expression by B cells has been shown to be an important maturation signal for CD169$^+$ macrophages in LNs (Phan et al., 2009). In the absence of B cell-derived LT, there is a dramatic loss of CD169$^+$ expression in the LN sub-capsular sinus (Iannacone et al., 2010; Moseman et al., 2012). RANK has been suggested to play a role in DC survival and longevity during the immune response (Josien et al., 1999, 2000; Kool et al., 2011), but the role of RANKL/RANK signaling in the development and function of MZM has not been studied.

Here, we have addressed the role of RANK signaling in macrophages and DCs during the priming and activation of mCTLs in the context of infection. We generated mice with targeted deletion of RANK (*Tnfrsf11a*) in DCs and demonstrate that RANK signaling is redundant for priming naive CD8 T cells but is specifically required for activation of mCTLs in response to viral infection. The role of RANK in mCTL activation is 2-fold; RANK expression in CD169$^+$ MZMs provides a niche for early viral replication during secondary infection. The loss of CD169$^+$ MZMs upon RANK deletion leads to impaired viral replication and the acquisition of viral antigen by cross-presenting DCs. However, RANK expression in DCs is also required to promote the recruitment and activation of pathogen-specific mCTLs. These studies reveal an important role for the RANKL/RANK signaling axis in the orchestration of protective immune responses in the spleen MZ and the host response to viral infection.

RESULTS

RANK Expression Does Not Affect DC Survival or Longevity

RANK is widely expressed among macrophages and DCs at the mRNA level (https://www.immgen.org; biogps.org). To determine the intrinsic roles of RANK signaling in these cells, we generated mice with a targeted deletion of RANK (*Tnfrsf11a*) in CD11c-expressing cells by intercrossing *Tnfrsf11a*$^{f/f}$ mice (Hanada et al., 2009) and Tg(*Itgax-Cre*) mice (Caton et al., 2007). Cre expression in these mice is targeted to DCs and certain macrophage populations that express CD11c, but not CD11c-negative monocyte/macrophage populations (Caton et al., 2007; Farrell et al., 2015). *Tnfrsf11a*$^{\Delta Itgax/\Delta Itgax}$ mice (*Tnfrsf11a*$^{\Delta Itgax}$) were born at normal Mendelian ratios and developed to adulthood, in contrast to mice with a germline deletion in RANK (*Tnfrsf11a*$^{-/-}$) that were severely runted at 3 weeks of age due to defects in tooth eruption and development (Li et al., 2000). We confirmed the deletion of RANK expression in CD11c$^+$ cells from *Tnfrsf11a*$^{\Delta Itgax}$ mice by flow cytometry (Figures 1A–1C). No perturbations in absolute numbers of myeloid or lymphoid cells were observed in naive *Tnfrsf11a*$^{\Delta Itgax}$ mice compared to littermate controls (data not shown).

Previous studies have suggested that RANK expression by DCs increased their survival and longevity and, thus, T cell priming during the immune response (Josien et al., 2000). Flow cytometry analysis revealed no differences in DC number in LNs or spleen from *Tnfrsf11a*$^{\Delta Itgax}$ mice compared to littermate controls at steady state (Figure 1D). Furthermore, the proportions of the major DC subsets—CD8α-type DC (DC1), CD11b-type DC (DC2), and pDC—remained unaltered (Figure 1E); detailed gating strategies are shown in Figure S1. DC maturation in steady state can be measured by the migration of tissue-resident DCs to draining LNs, which is also associated with the upregulation of RANK expression (Baratin et al., 2015; Dalod et al., 2014); however, accumulation of migratory DCs in cutaneous LNs was also not altered in *Tnfrsf11a*$^{\Delta Itgax}$ mice (Figure 1F). To further test the intrinsic role of RANK in DC survival and homeostasis, we performed competitive bone marrow (BM) chimera experiments. BM cells from *Tnfrsf11a*$^{\Delta Itgax}$ and *Tnfrsf11a*$^{f/f}$ mice (CD45.2$^+$) were mixed 1:1 with BM cells from CD45.1$^+$/CD45.2$^+$ mice and then adoptively transferred to lethally irradiated CD45.1$^+$ recipients. Eight weeks following adoptive transfer, chimeric mice were exposed to the thymidine analog bromodeoxyuridine (BrdU) in drinking water for 8 days, after which LNs were harvested for flow-cytometric analysis of BrdU incorporation by DCs. The proportions of both resident and migratory cells and incorporation of BrdU was equal among DCs derived from *Tnfrsf11a*$^{\Delta Itgax}$ or *Tnfrsf11a*$^{f/f}$ BM cells (Figure 1G), indicating no intrinsic defect in DC survival or longevity in steady state. To test the role of RANK in DC homeostasis during inflammation, we used topical application of the TLR7/8 agonist imiquimod (Aldara cream containing 5% Imiquimod; IMQ), a clinically used immune adjuvant (van der Fits et al., 2009). IMQ treatment dramatically increased the number of RANK$^+$ DCs in draining LNs and significantly increased the level of RANK expression (Figure 1H). However, when we compared the total numbers of DCs in LNs after IMQ treatment, there was no difference in the absence of RANK expression (Figures 1I and 1J). These data clearly showed that RANK deletion did not affect the survival of DCs in steady state or during inflammation.

RANK Expression in CD11c$^+$ Cells Regulates mCTL Activation in Response to Viral Infection

To test the role of RANK expression in CD11c$^+$ cells for T cell priming during infection, we used a recombinant strain of the intracellular bacteria *Listeria monocytogenes* engineered to express ovalbumin (Lm-OVA) (Bajénoff et al., 2010) and an OVA-expressing strain of vesicular stomatitis virus (VSV-OVA) (Kim et al., 1998). We infected *Tnfrsf11a*$^{f/f}$ and *Tnfrsf11a*$^{\Delta Itgax}$ mice intravenously (i.v.) with 10^4 colony-forming units (CFUs) of Lm-OVA or 10^5 plaque-forming units (PFUs) of VSV-OVA and measured the priming of pathogen-specific CD8 T cells. Eight days after primary infection, the expansion of OVA-specific CD8 T cells was measured in blood by flow cytometry, using OVA-specific major histocompatibility complex (MHC) class I tetramers (H2Kb^{SIINFEKL}; Tet). The frequency of Tet$^+$ CD8

(H) Proportion of RANK$^+$ DCs in CLN from wild-type mice with or without IMQ treatment.
(I) Proportion of RANK$^+$ DCs in CLN from *Tnfrsf11a*$^{f/f}$ and *Tnfrsf11a*$^{\Delta Itgax}$ mice after IMQ treatment; representative FACS plots are shown.
(J) Quantification of total DC numbers in CLN from *Tnfrsf11a*$^{f/f}$ and *Tnfrsf11a*$^{\Delta Itgax}$ mice 2 days after IMQ treatment.
Data are represented as mean ± SEM of n ≥ 3 and are representative of at least 2 independent experiments.

T cells and intracellular interferon (IFN)γ production after stimulation with cognate peptide was comparable between *Tnfrsf11a*$^{f/f}$ and *Tnfrsf11a*$^{\Delta Itgax}$ mice (Figures 2A and 2B). Similarly, granzyme B (GrzB) expression by OVA-specific CD8 T cells was unaltered in *Tnfrsf11a*$^{\Delta Itgax}$ mice (Figure S2A). These data demonstrate that RANK expression by CD11c^{+} cells was not required for priming naive CD8 T cells. Conventional DCs are characterized by their unique roles in priming naive T cells (Steinman, 2008), but CD11c-expressing cells have also been shown to be required for triggering mCTLs upon secondary infection (Zammit et al., 2005). To test the role of RANK expression during mCTL activation, we immunized cohorts of *Tnfrsf11a*$^{f/f}$ and *Tnfrsf11a*$^{\Delta Itgax}$ mice with Lm-OVA, VSV-OVA, or IMQ/OVA and waited 2 months for the contraction of effector T cell responses and the generation of mCTLs. We then challenged mice with VSV-OVA or Lm-OVA to trigger the activation of mCTLs (Figures 2C–2F); Lm-OVA or IMQ/OVA immunized *Tnfrsf11a*$^{\Delta Itgax}$ mice showed a significant impairment in mCTL activation upon infection with VSV-OVA, compared with littermate controls, as indicated by reduced expansion of OVA-specific CD8 T cells in the spleen (Figures 2C, 2D, and S2B). In contrast, Lm-OVA or VSV-OVA immunized *Tnfrsf11a*$^{\Delta Itgax}$ mice showed no defect in mCTL activation in response to infection with Lm-OVA (Figures 2E, 2F, and S2C). These data showed that RANK expression in CD11c^{+} cells was specifically required for triggering mCTLs in response to VSV infection and was redundant for mCTL activation by Lm. We observed no differences in the recall of mCTLs harvested from IMQ/OVA or Lm-OVA immunized *Tnfrsf11a*$^{\Delta Itgax}$ mice *ex vivo* in the presence of cognate peptide (Figure S3), indicating that development of pathogen-specific mCTLs was not affected by RANK deletion in CD11c-expressing cells. To determine whether RANK expression in antigen-presenting cells affected the programming of mCTLs during the primary immune response, we infected congenic CD45.1^{+} mice with Lm-OVA and, 3 weeks later, adoptively transferred splenic T cells (CD4 and CD8) to naive *Tnfrsf11a*$^{f/f}$ and *Tnfrsf11a*$^{\Delta Itgax}$ mice (CD45.2^{+}). Based on tetramer staining prior to adoptive transfer, we estimate that approximately 5,000 OVA-specific effector-mCTL were injected per mouse. We then waited a further 7 weeks and challenged mice with VSV-OVA or Lm-OVA to activate adoptively transferred mCTLs (CD45.1^{+}) (Figure 2G). Subsequent flow cytometry analysis of VSV-OVA-challenged mice showed a clear defect in expansion of CD45.1^{+} mCTLs in *Tnfrsf11a*$^{\Delta Itgax}$ mice compared to littermate controls (Figures 2H and S4A); however, there was no defect in the priming of naive endogenous CD8 T cells (CD45.2^{+}). In contrast, mice challenged with Lm-OVA showed no defect in the expansion or activation of CD45.1^{+} mCTLs (Figures 2I and S4B) and the priming of naive endogenous CD8 T cells (CD45.2^{+}). These experiments confirmed that RANK expression in CD11c^{+} cells is required for the triggering of mCTLs upon infection with VSV and not the priming or functional programming of memory cells during the primary immune response.

RANK Is Required for Accumulation of CD169^{+} MZMs

After intravenous infection, mCTLs are activated in the spleen MZ and inter-follicular regions of the red pulp, whereas naive T cells are primed in the follicular T cell zone of the white pulp (Schenkel et al., 2014). Furthermore, VSV and Lm have been reported to occupy distinct cellular niches for replication within the spleen, with VSV being restricted to CD169^{+} MZM (Honke et al., 2011), whereas Lm specifically targets migratory DC1 and replicates in the T cell zone of the white pulp (Edelson et al., 2011). RANK ligand (RANKL; TNFSF11) has also been shown to be constitutively expressed by MRCs that line the spleen MZ (Jarjour et al., 2014; Katakai et al., 2008), in direct contact with CD169^{+} MZMs. Given that VSV specifically targets CD169^{+} MZMs and the distinctive expression of RANKL in this niche, we reasoned that RANK signaling may have a role in the development or function of these cells. To test this hypothesis, we first confirmed expression of RANKL by MRCs in the spleen MZ by confocal microscopy, which was unaltered in *Tnfrsf11a*$^{\Delta Itgax}$ mice (Figure 3A). We then assessed the impact of RANK deletion on MZMs; there was an obvious reduction in non-specific esterase staining in the MZ of spleens from *Tnfrsf11a*$^{\Delta Itgax}$ mice compared to littermate controls—a distinctive marker of metallophilic MZMs (Figure 3B). Furthermore, there was also a significant reduction in CD169^{+} cells in the MZ of *Tnfrsf11a*$^{\Delta Itgax}$ mice, whereas CD209b (SIGN-R1)$^{+}$ macrophages in the outer MZ were unaffected (Figures 3C and 3D). The close proximity of RANKL^{+} MRCs and CD169^{+} MZMs suggested that intrinsic RANK signaling could be required to maintain CD169-expressing cells. Previous studies have shown that CD11c is expressed by at least a subset of CD169^{+} cells in the MZ (Farrell et al., 2015; Probst et al., 2005); to confirm targeting of CD169^{+} cells in CD11c-Cre mice, we crossed these mice to *Rosa26-lsl-tdRFP* reporter mice (Luche et al., 2007). Analysis of spleen sections from these mice by confocal microscopy showed red fluorescent protein (RFP) expression in a subset of CD169^{+} cells in the MZ, as well as cells throughout the white pulp and red pulp of the spleen (Figure 3E), confirming efficient Cre recombinase activity in these cells and, thus, targeting of these cells in *Tnfrsf11a*$^{\Delta Itgax}$ mice. Furthermore, an analysis of spleen sections from Tg(*Itgax-EYFP*) mice, which express yellow fluorescent protein (YFP) from the CD11c promoter, clearly showed that a subset of CD169^{+} cells in the MZ actively expressed CD11c (Figure 3F). These data suggested that intrinsic RANK signaling was required for the maintenance of CD11c^{+} CD169^{+} MZMs.

RANK-Dependent MZMs Provide a Niche for VSV Replication

Since CD169^{+} MZMs have previously been shown to provide a restricted replicative niche for VSV (Honke et al., 2011), we reasoned that loss of RANK-dependent MZMs could affect VSV replication. To test this hypothesis, we measured viral titers in spleens harvested from *Tnfrsf11a*$^{\Delta Itgax}$ and *Tnfrsf11a*$^{f/f}$ mice 7 hr after infection with VSV-OVA. There was a significant reduction in splenic VSV titers from *Tnfrsf11a*$^{\Delta Itgax}$ mice compared to littermate controls (Figure 4A), which correlated with reduced expression of virus-encoded OVA, determined by qRT-PCR with RNA isolated from total splenocytes (Figure 4B). These data showed that RANK signaling in CD11c-expressing cells promotes early viral replication and expression of viral antigens in the spleen. To confirm the restricted replication of VSV in CD169^{+} cells *in situ*, we used a recombinant strain of VSV expressing GFP (VSV-GFP) and performed confocal microscopy

OPEN
ACCESS
CellPress

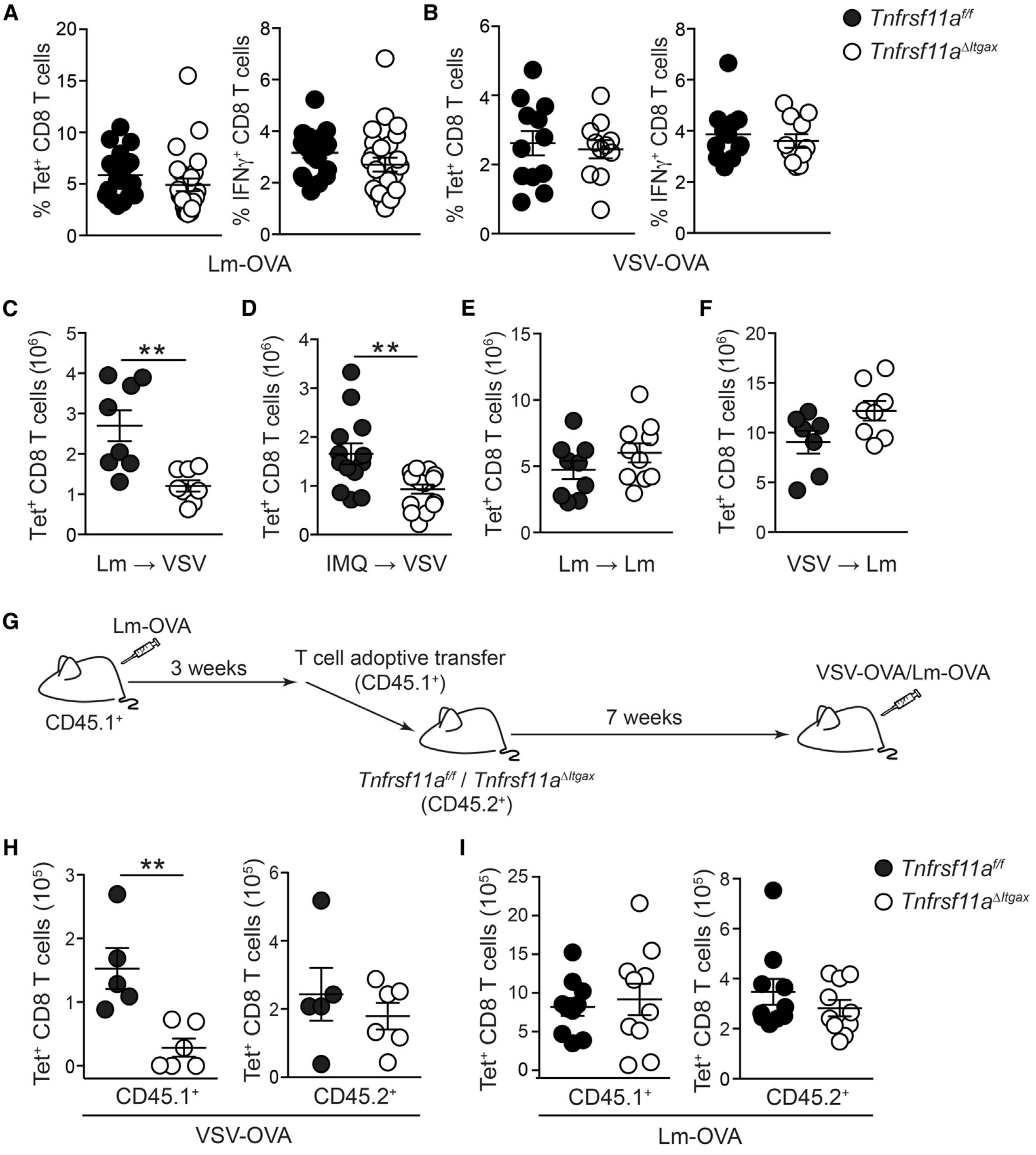

Figure 2. RANK Expression by CD11c$^+$ Cells Regulates mCTL Activation in Response to Viral Infection

(A and B) Expansion of endogenous OVA-specific CD8 T cells (Tet-H2Kb^{SIINFEKL}; Tet$^+$) was measured in peripheral blood from *Tnfrsf11a*$^{f/f}$ and *Tnfrsf11a*$^{\Delta Itgax}$ mice 1 week after infection i.v. with 1×10^4 CFUs of Lm-OVA (A) or 1×10^5 PFUs of VSV-OVA (B). IFNγ production was measured by intracellular cytokine staining (ICS) after *ex vivo* stimulation with cognate peptide (SIINFEKL).

(C–F) Cohorts of *Tnfrsf11a*$^{f/f}$ and *Tnfrsf11a*$^{\Delta Itgax}$ mice were immunized with Lm-OVA, IMQ/OVA, or VSV-OVA; (C) Lm → VSV, (D) IMQ → VSV, (E) Lm → Lm, and (F) VSV → Lm. 2 months later, mice were challenged with either VSV-OVA (C and D) or Lm-OVA (E and F). 5 days later, expansion of OVA-specific mCTLs (CD44$^+$ Tet$^+$) was measured in spleen by flow cytometry.

(G–I) Shown in (G): experimental strategy for the adoptive transfer of mCTLs. CD45.1$^+$ mice were immunized with Lm-OVA; 3 weeks later, CD4 and CD8 T cells were isolated from spleen and adoptively transferred to cohorts of naive *Tnfrsf11a*$^{f/f}$ and *Tnfrsf11a*$^{\Delta Itgax}$ mice. A further 7 weeks after T cell transfer, mice were infected with VSV-OVA (H) or Lm-OVA (I); activation of adoptively transferred mCTLs (CD44$^+$, CD45.1$^+$) and priming of endogenous naive CD8 T cells (CD45.2$^+$) were measured simultaneously in spleen 5 days after challenge as expansion of Tet$^+$ cells.

Data are represented as mean ± SEM, and statistical analysis was performed with the Mann-Whitney test; $^{**}p < 0.01$.

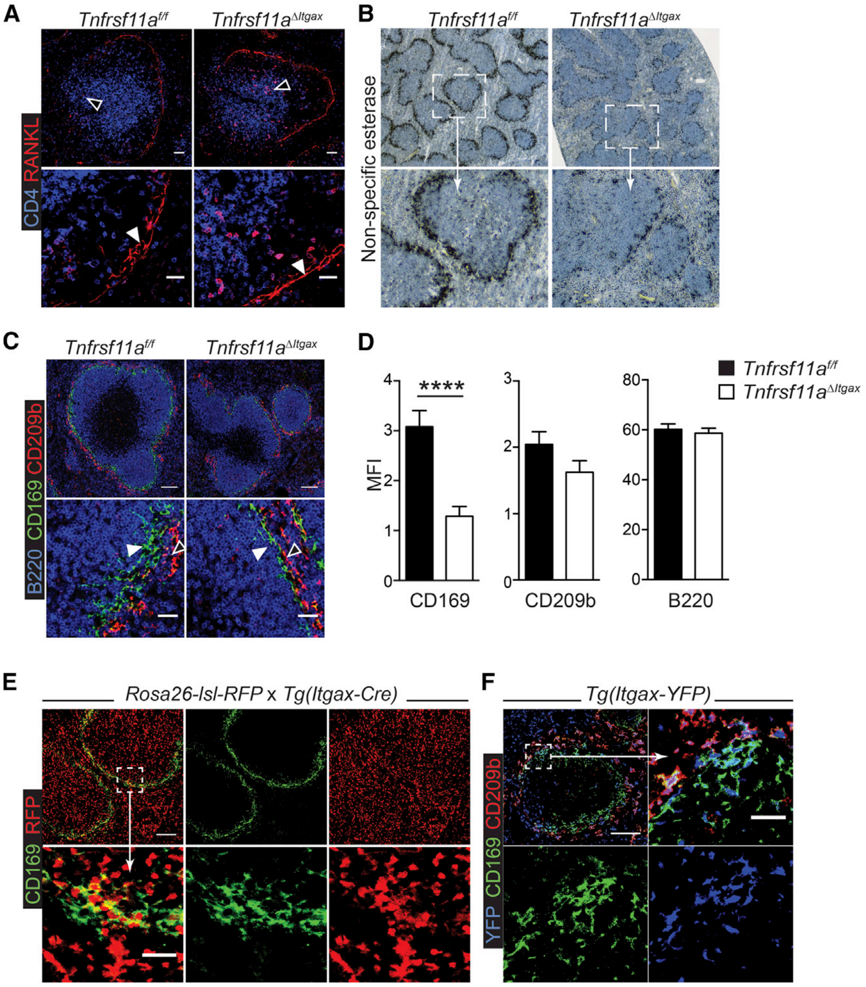

Figure 3. RANK Is Required for CD11c$^+$ CD169$^+$ MZMs

(A–D) Immunohistochemical analysis of spleen sections from *Tnfrsf11a*$^{f/f}$ and *Tnfrsf11a*$^{\Delta Itgax}$ mice analyzed by confocal microscopy. (A) RANKL staining (red) of marginal reticular stromal cells (MRCs) in the spleen marginal zone (MZ); CD4 staining for T cells is in blue. (B) Non-specific esterase staining of spleen sections. (C) CD169 (green; filled arrowheads) and CD209b staining (red; open arrowheads); B cells are stained with B220 (blue). (D) Quantification of CD169, CD209b, and B220 staining in the spleen MZ of *Tnfrsf11a*$^{f/f}$ and *Tnfrsf11a*$^{\Delta Itgax}$ mice. Data are represented as the mean intensity (±SEM) within the MZ from n ≥ 3 mice.

(E) Spleen sections from *Rosa26-LSL-tdRFP* × Tg(*Itgax-Cre*) mice showing Cre-recombinase activity in CD169$^+$ cells; red indicates tdRFP, and green indicates CD169.

(F) Spleen sections from Tg(*Itgax-EYFP*) mice showing CD11c expression in CD169$^+$ cells; blue indicates YFP, green indicates CD169, and red indicates CD209b. Scale bars: 100 μm in the upper panels and 25 μm in the lower panels. Representative micrographs are shown from at least 2 independent experiments.

Statistical analysis was performed with the Mann-Whitney test; ****$p < 0.0001$.

(Iannacone et al., 2010). As expected, GFP expression co-localized with CD169$^+$ cells in the spleen MZ after infection with VSV-GFP, and there was a drastic reduction of GFP$^+$ cells in spleens harvested from *Tnfrsf11a*$^{\Delta Itgax}$ mice compared to littermate controls (Figures 4C and 4D); this correlated with a significant reduction in total VSV-GFP titers (Figure 4E). Recent studies have shown that cross-presenting DC1 cells, which localize to the MZ and interfollicular regions of the spleen, are required for full activation of mCTLs after VSV infection, as well as other pathogens, including Lm (Alexandre et al., 2016). Flow cytometry analysis revealed acquisition of VSV-GFP by splenic DCs—specifically, the DC1 subset (Figure 4F). Furthermore, in keeping with reduced VSV replication, significantly lower levels of VSV-GFP were detected in DCs from *Tnfrsf11a*$^{\Delta Itgax}$ mice (Figure 4G). Total numbers of splenic DC1 cells were not altered in *Tnfrsf11a*$^{\Delta Itgax}$ mice (Figure 1), which suggested that the reduced acquisition of VSV was due to reduced replication in CD169$^+$ MZMs.

To confirm that the reduction in VSV replication was due to the loss of RANK signaling in CD11c$^+$ MZMs and not DCs, we crossed *Tnfrsf11a*$^{f/f}$ mice with *Lyz2*$^{cre/+}$ mice (Clausen et al., 1999), which express Cre recombinase in macrophages but not conventional DCs (Baratin et al., 2015). In keeping with an intrinsic role for RANK signaling in CD169$^+$ MZMs, *Tnfrsf11a*$^{\Delta Lyz2}$ mice also showed a significant loss of CD169$^+$ cells in the MZ, compared to littermate controls (Figures 5A–5C and S5A). Furthermore, VSV replication was significantly impaired in *Tnfrsf11a*$^{\Delta Lyz2}$ mice compared to littermates (Figure 5D). However, the loss of MZMs and reduced viral replication in *Tnfrsf11a*$^{\Delta Lyz2}$ mice was not sufficient to impair either the priming or activation of mCTLs in response to VSV infection (Figures 5E and 5F). We confirmed that RANK expression by DCs was retained in *Tnfrsf11a*$^{\Delta Lyz2}$ mice, as opposed to *Tnfrsf11a*$^{\Delta Itgax}$ mice (Figure S5B), suggesting that defective mCTL activation upon RANK deletion in CD11c$^+$ cells was likely due to the expression of RANK by DCs. These data showed that RANK expression by MZMs maintains a replicative niche for VSV and suggested that RANK expression by DCs was additionally required to promote mCTL activation.

Collaboration of MZM and DC1 for Activation of mCTLs

Recent studies have shown that DC1 cells are required for full activation of mCTLs in response to several pathogens, including VSV (Alexandre et al., 2016). We observed a striking increase in RANK expression by splenic DCs upon VSV infection of immunized mice (Figure 6A), demonstrating that RANK expression is upregulated by DCs during mCTL activation. RANK expression was also strongly induced by DCs in mice treated with the TLR7/8 agonist IMQ (Figure 1H), indicating that induction of RANK expression on DCs was a direct consequence of viral

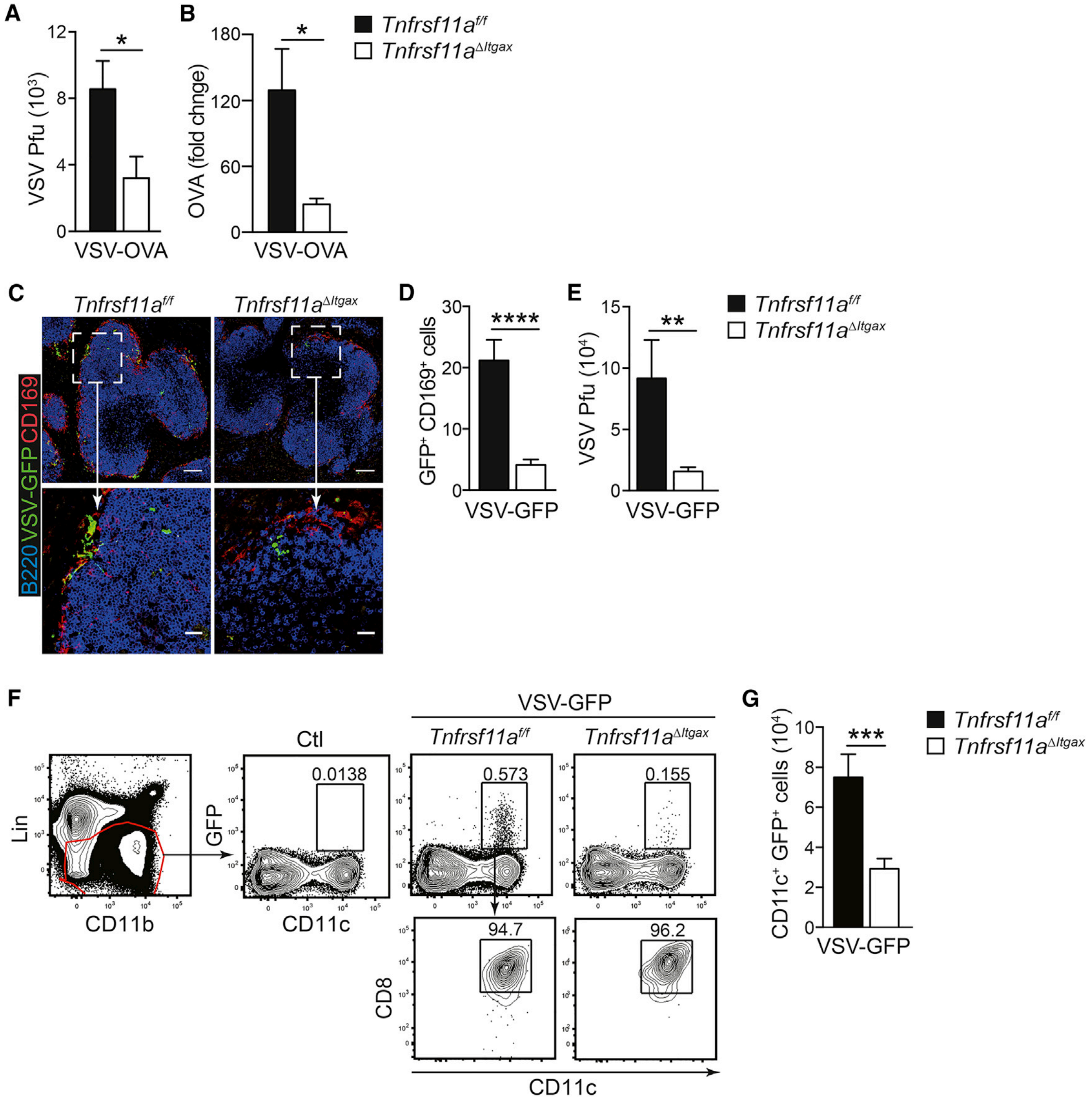

Figure 4. RANK-Dependent MZMs Provide a Niche for VSV Replication

(A and B) Lm-OVA-immunized mice were infected i.v. with VSV-OVA, and spleens were harvested after 7 hr for analysis of viral replication. (A) Splenic titers of VSV-OVA from immunized *Tnfrsf11a*$^{f/f}$ and *Tnfrsf11a*$^{\Delta Itgax}$ mice. (B) qRT-PCR analysis of OVA expression in total splenocytes.

(C and D) Lm-OVA-immunized *Tnfrsf11a*$^{f/f}$ and *Tnfrsf11a*$^{\Delta Itgax}$ mice were challenged with VSV-GFP. (C) Confocal microscopy of spleen sections 7 hr after infection with VSV-GFP; viral replication (green) is shown in CD169^{+} cells in the MZ (red); B cells are stained with B220 (blue). Scale bars: 100 μm in the upper panels and 25 μm in the lower panels. Representative micrographs are shown from at least 2 independent experiments. (D) Quantification of GFP^{+} CD169^{+} cells in the MZ. (E) Splenic titers of VSV-GFP from *Tnfrsf11a*$^{f/f}$ and *Tnfrsf11a*$^{\Delta Itgax}$ mice 7 hr after infection.

(F and G) Flow cytometry analysis of GFP expression in CD8α^{+} splenic DCs from Lm-OVA-immunized *Tnfrsf11a*$^{f/f}$ and *Tnfrsf11a*$^{\Delta Itgax}$ mice 7 hr after VSV-GFP infection. Gating strategy (F) and quantification (G) are shown.

Representative FACS plots are shown, and graphs represent mean ± SEM of n ≥ 6. Statistical analysis was performed with the Mann-Whitney test. *$p < 0.05$; **$p < 0.01$; ****$p < 0.0001$.

infection. To directly test the effects of RANK signaling on activation of CD8 T cells by DCs, we performed co-culture experiments with DCs derived from *Tnfrsf11a*$^{\Delta Itgax}$ and *Tnfrsf11a*$^{f/f}$ mice and RANKL-expressing stromal cells; in these experiments, RANKL significantly increased the expansion of antigen-specific CD8 T cells and IFNγ production in a RANK-dependent manner (Figure S6), demonstrating that RANKL/RANK signaling in DCs can enhance CD8 T cell activation in response to exogenous antigen.

We next assessed the role of RANK in DC activation *in vivo* during mCTL activation by VSV. mCTLs patrol the spleen MZ during recall responses and rapidly form clusters with DCs (Bajénoff et al., 2010; Schenkel et al., 2014). The early recruitment of mCTLs is driven by the IFNγ-dependent induction of CXCL9

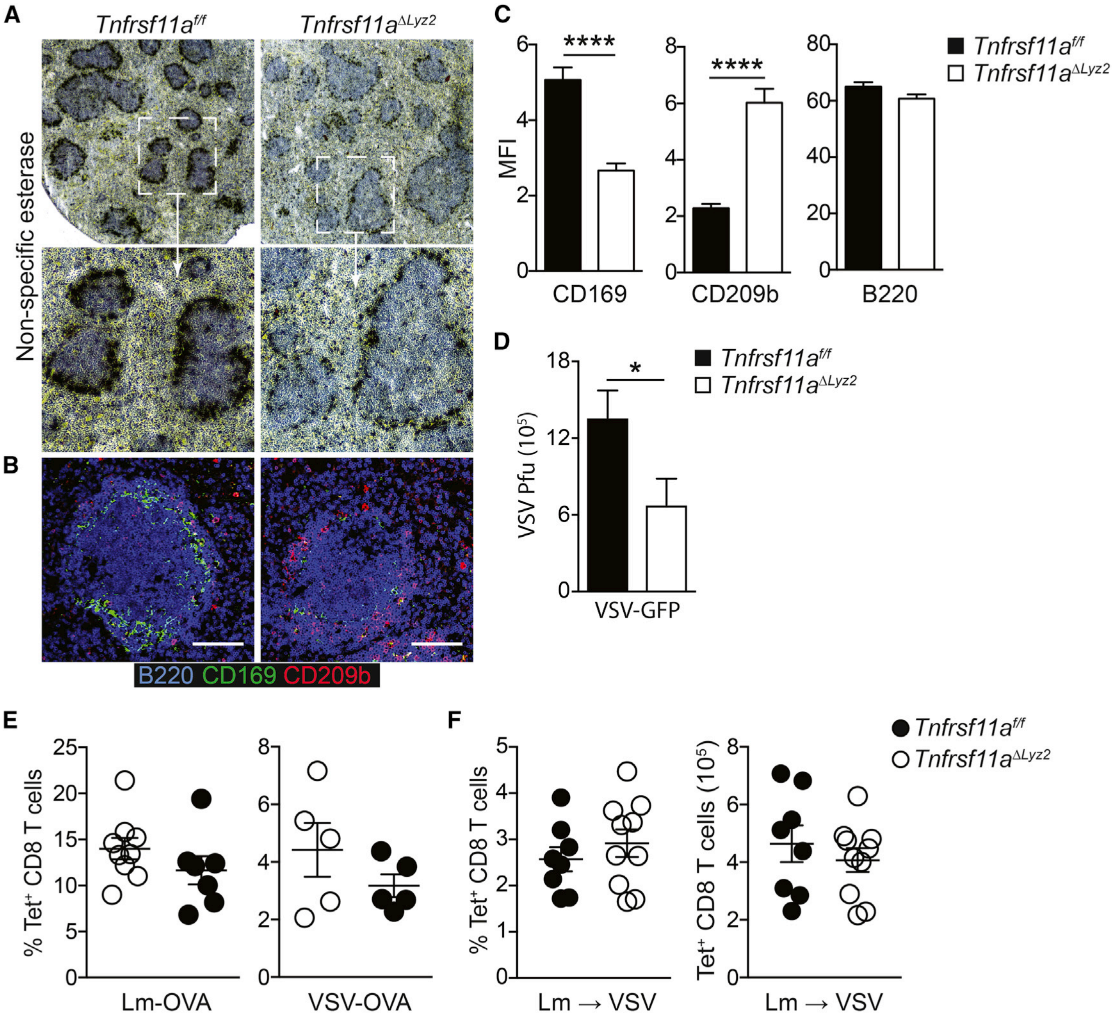

Figure 5. RANK-Dependent MZMs Are Not Sufficient for mCTL Activation

(A–C) Immunohistochemical analysis of spleen sections from *Tnfrsf11a*$^{f/f}$ and *Tnfrsf11a*$^{\Delta Lyz2}$ mice analyzed by confocal microscopy; (A) Non-specific esterase staining of spleen sections. (B) CD169 (green; filled arrowheads) and CD209b staining (red; open arrowheads); B cells are stained with B220 (blue). Scale bars: 100 μm. Representative micrographs are shown from at least 2 independent experiments. (C) Quantification of CD169, CD209b, and B220 staining in the spleen MZ of *Tnfrsf11a*$^{f/f}$ and *Tnfrsf11a*$^{\Delta Lyz2}$ mice; data are represented as the mean intensity (±SEM) within the MZ from n ≥ 3 mice.

(D) *Tnfrsf11a*$^{f/f}$ and *Tnfrsf11a*$^{\Delta Lyz2}$ mice were infected i.v. with 10^6 PFUs of VSV-GFP, and spleens were harvested after 7 hr for analysis of viral replication.

(E) Expansion of OVA-specific CD8 T cells (Tet+) was measured in blood from *Tnfrsf11a*$^{f/f}$ and *Tnfrsf11a*$^{\Delta Lyz2}$ mice 8 days after infection i.v. with Lm-OVA or VSV-OVA.

(F) Cohorts of *Tnfrsf11a*$^{f/f}$ and *Tnfrsf11a*$^{\Delta Lyz2}$ mice were immunized with Lm-OVA and, 2 months later, challenged i.v. with VSV-OVA; expansion of OVA-specific mCTLs ($CD44^+$ Tet^+) was measured 5 days later in spleen by flow cytometry.

Graphs represent mean ± SEM of n = 5. Statistical analysis was performed with Mann-Whitney test. *$p < 0.05$; ****$p < 0.0001$.

expression by DCs (Alexandre et al., 2016; Sung et al., 2012). We measured IFNγ and CXCL9 expression in spleen from Lm-OVA-immunized *Tnfrsf11a*$^{f/f}$ and *Tnfrsf11a*$^{\Delta Itgax}$ mice 7 hr after challenge with VSV-OVA. Both IFNγ and CXCL9 were significantly reduced at this early time point in *Tnfrsf11a*$^{\Delta Itgax}$ mice upon secondary challenge with VSV-OVA, but not after primary infection with VSV (Figure 6B), indicating that early triggering of IFNγ production by mCTLs and subsequent induction of CXCL9 expression were impaired in the absence of RANK. Analysis of CXCL9 expression in spleen sections from VSV-challenged mice by confocal microscopy confirmed the specific induction of CXCL9 expression in DCs within the MZ, which was absent in *Tnfrsf11a*$^{\Delta Itgax}$ mice (Figure 6C). Furthermore, flow cytometry analysis showed a significant reduction in the frequency of $CXCL9^+$ DCs in *Tnfrsf11a*$^{\Delta Itgax}$ mice upon VSV challenge, compared with littermate controls (Figure 6D). These data suggested that early viral replication in RANK-dependent MZMs provided an easily accessible source of antigen and inflammatory signals for triggering mCTLs by DCs, which subsequently drives CXCL9 induction to perpetuate the memory T cell response. To further test this hypothesis, we sought to visualize the recruitment of mCTLs in the spleen of mice after VSV

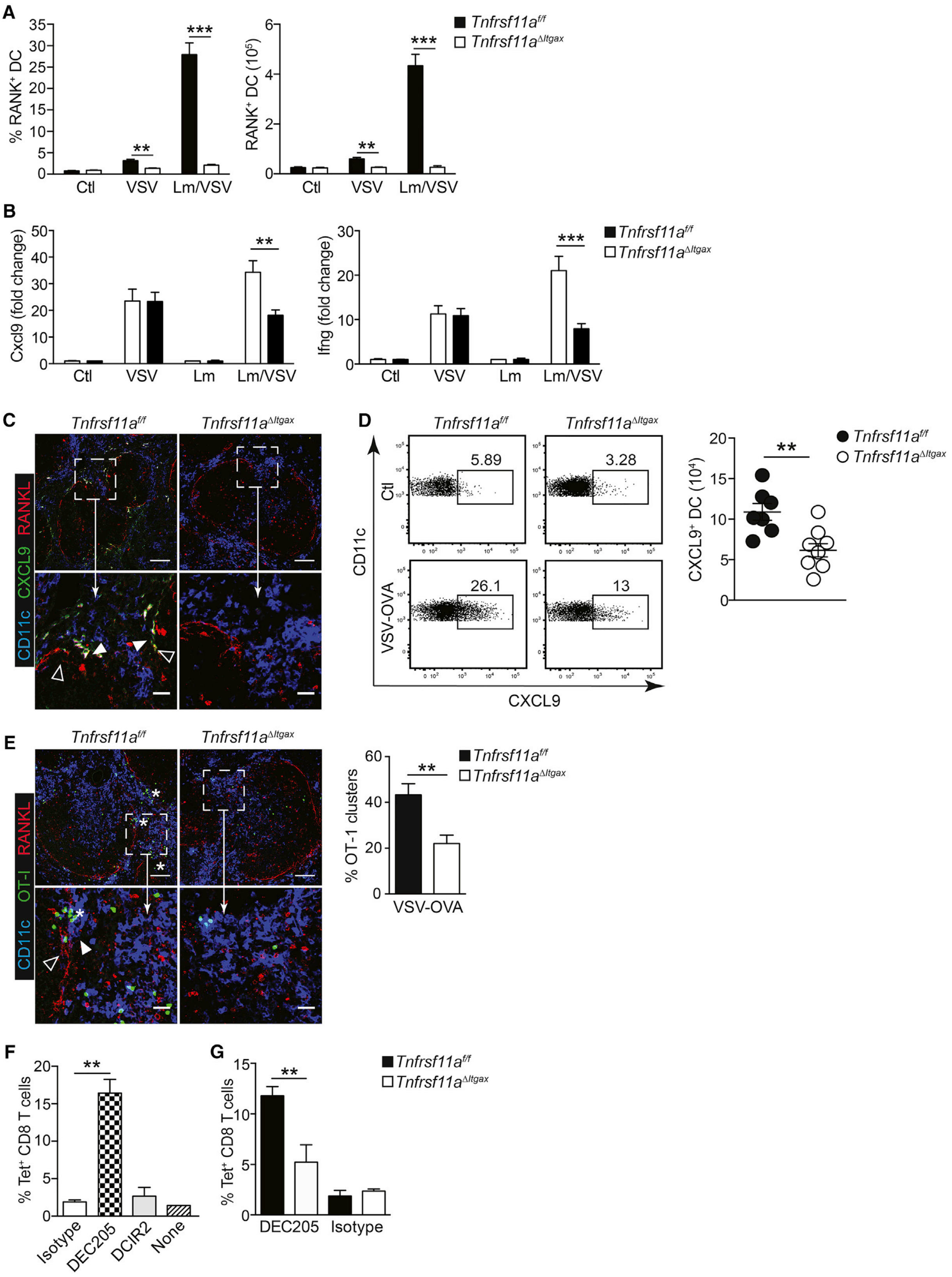

(legend on next page)

infection. To track the mobilization of mCTLs, we adoptively transferred OVA-specific CD8 T cells from OT-I-GFP mice; 2 months after immunization with Lm-OVA, *Tnfrsf11a*$^{f/f}$ and *Tnfrsf11a*$^{\Delta Itgax}$ mice that had received OT-I-GFP cells were infected with VSV-OVA, and the spleens were harvested after 7 hr. We performed confocal microscopy on spleen sections to localize OT1-GFP cells. Mobilization of memory OT-I-GFP cells to the splenic MZ and interfollicular regions could clearly be seen upon VSV-OVA challenge of immunized mice (Figure 6E). Furthermore, as expected, the recruitment of GFP-expressing OT-I cells was reduced significantly in *Tnfrsf11a*$^{\Delta Itgax}$ mice, which correlated with the impaired induction of IFNγ and CXCL9 expression (Figure 6B). These data demonstrate that DC-mediated triggering of mCTLs during secondary infection is RANK dependent.

To directly test the contribution of RANK signaling in DCs to mCTL activation, we used OVA-conjugated monoclonal antibodies to target antigen specifically to DCs in the context of VSV infection. Lm-OVA-immunized *Tnfrsf11a*$^{f/f}$ or *Tnfrsf11a*$^{\Delta Itgax}$ mice were infected with wild-type VSV (not expressing OVA), and 1 hr later, OVA-conjugated anti-DEC205 (CD205) or anti-DCIR2 (CLEC4A) was injected intraperitoneally (i.p.) to target OVA specifically to DC1 (CD205$^+$) or DC2 (CLEC4A$^+$), respectively. Five days later, endogenous OVA-specific mCTL expansion was measured in spleen by flow cytometry. In wild-type mice, we observed a strong activation of mCTL when the OVA antigen was targeted to DC1, but not DC2 (Figure 6F), confirming the specific role of DC1 in mCTL activation in this context. However, recall of mCTLs was still significantly impaired in *Tnfrsf11a*$^{\Delta Itgax}$ mice when antigen was targeted directly to DC1 (Figure 6G), confirming that RANK signaling in DCs contributes to mCTL activation independently of antigen acquisition from MZM.

In summary, we demonstrate that RANK signaling plays critical roles in viral replication and mCTL activation in the spleen MZ. The role of RANK is 2-fold; first, RANK expression is required in CD169$^+$ MZMs to provide a niche for early viral replication. Second, RANK signaling in DCs is additionally required to propagate mCTL activation. The selective expression of RANKL by MRCs in the spleen MZ is likely to dictate the restricted roles of RANK in mCTL activation during infection with pathogens that occupy this specific niche. These studies reveal an important role for the RANKL/RANK signaling axis in the orchestration of protective immunity during viral infection.

DISCUSSION

Macrophages and DCs are strategically located in the MZ of the spleen at the interface with blood circulation where they can capture bloodborne microbes and antigens. CD169$^+$ MZMs have been shown to play important roles in trafficking antigens to B cells and as a replicative niche for certain pathogens. Both CD169$^+$ MZMs and DCs have also been suggested to contribute to cell-mediated immunity; however, little is known about the mechanisms that regulate the functions of these cells during the immune response to bloodborne pathogens and their relative contributions to protective immunity. The RANKL/RANK signaling axis between LTis, of hematopoietic origin, and LTos is critical for lymphoid organogenesis. In the mature spleen, RANKL remains constitutively expressed by MRCs, which line the marginal sinus (Jarjour et al., 2014; Katakai et al., 2008), but the role of RANKL/RANK signaling in the orchestration of immune responses in this niche has not previously been addressed.

We generated mice with targeted deletion of RANK (*Tnfrsf11a*) in macrophages and DCs. Although previous reports had suggested a role for RANK in DC survival and T cell priming (Josien et al., 1999, 2000; Kool et al., 2011), we found no role for RANK expression in DC homeostasis or CD8 T cell priming in the context of infection. However, deletion of RANK expression resulted in a significant defect in CD169$^+$ MZMs. Previous reports have shown that LT, another TNF family member, expressed by B cells provides a maturation signal for CD169$^+$ macrophages in the LN sub-capsular sinus (Phan et al., 2009). However, a role of RANKL/RANK signaling in the development or function of these cells has never been shown. We found no defects in B cell homeostasis after deletion of RANK expression in CD11c$^+$ cells (data not shown), indicating that the role of RANK in the maintenance of CD169$^+$ MZM is complementary to B cell-derived LT.

Figure 6. Collaboration of MZMs and DC1 for Activation of mCTLs

(A) Frequency of RANK$^+$ DC in spleen 7 hr after primary or secondary VSV challenge in *Tnfrsf11a*$^{f/f}$ and *Tnfrsf11a*$^{\Delta Itgax}$ mice.

(B) Naive or Lm-OVA-immunized *Tnfrsf11a*$^{f/f}$ and *Tnfrsf11a*$^{\Delta Itgax}$ mice were infected with VSV-OVA; 7 hr after infection, induction of CXCL9 and IFNγ mRNA expression were measured in total splenocytes by qRT-PCR. Data are expressed as fold induction compared to uninfected mice (Ctl) and are indicated as the mean $\pm$ SEM of n = 7–9.

(C) Localization of CXCL9$^+$ DCs (green/white; filled arrowheads) in the spleen MZ, marked by RANKL staining (red/pink; open arrowheads), in Lm-OVA-immunized *Tnfrsf11a*$^{f/f}$ and *Tnfrsf11a*$^{\Delta Itgax}$ mice 7 hr after infection with VSV-OVA.

(D) CXCL9 induction in splenic DCs was measured by intracellular flow cytometry; representative FACS plots are shown, and graph indicates mean $\pm$ SEM.

(E) OT-I-GFP cells were adoptively transferred to cohorts of *Tnfrsf11a*$^{f/f}$ and *Tnfrsf11a*$^{\Delta Itgax}$ mice prior to immunization with Lm-OVA. 2 months later, mice were infected with VSV-OVA, and spleens were collected after 7 hr for visualization of mCTL mobilization by confocal microscopy; recruitment of memory OT-I-GFP cells (green; asterisk) in the spleen MZ is indicated, juxtaposed to RANKL$^+$ MRCs (red; open arrowheads) and CD11c$^+$ DCs (blue; closed arrowheads). Scale bars: 100 μm in the upper panels and 25 μm in the lower panels. Representative micrographs are shown from at least 2 independent experiments. Bar graph indicates quantification of OT-I-GFP clusters in *Tnfrsf11a*$^{f/f}$ and *Tnfrsf11a*$^{\Delta Itgax}$ mice 7 hr after infection with VSV-OVA.

(F) Wild-type mice were immunized with Lm-OVA and, 4 months later, challenged i.v. with wild-type VSV and administration i.p. of either OVA-conjugated anti-DEC205, anti-DCIR2, or isotype-control antibody. Five days later, endogenous OVA-specific mCTL (CD44$^+$ Tet$^+$) expansion was measured in spleen by flow cytometry.

(G) Lm-OVA-immunized *Tnfrsf11a*$^{f/f}$ and *Tnfrsf11a*$^{\Delta Itgax}$ mice were challenged with wild-type VSV with OVA-conjugated anti-DEC205 or isotype-control antibody; mCTL expansion was measured in spleen after 5 days.

Graphs represent mean $\pm$ SEM of n = 2–6 for isotype control and n = 5–7 for the other groups. Statistical analysis was performed with the Mann-Whitney test. **$p < 0.01$; ***$p < 0.005$.

The overlapping functions of RANKL and LT in the regulation of CD169[+] MZMs is reminiscent of the RANKL/LT-mediated signaling cross-talk between LTi and LTo cells during lymphoid organogenesis; in this case, stromal LTo cells provide RANKL signals to amplify LT-dependent CXCL13 expression and formation of the developing LN (Brendolan and Caamaño, 2012). RANKL-expressing MRCs could be considered the adult equivalent of LTos (Katakai et al., 2008); thus, in mature lymphoid organs, RANKL expressed by MRCs may have both direct effects on CD169[+] MZM through intrinsic RANK signaling and indirect effects through the upregulation of LT expression on B cells.

Despite normal T cell priming, our data show that RANK expression in CD11c[+] cells is specifically required for mCTL activation in response to VSV infection and not the bacterial pathogen Lm, which reflects the loss of RANK-dependent CD169[+] MZMs that provide a replicative niche for VSV. The lack of effect on naive T cell priming is consistent with previous studies showing that splenic MZMs are not required for T cell priming in the context of VSV infection (Ciavarra et al., 1997; Junt et al., 2006). This may reflect the differential localization of naive and memory T cells in the spleen. Memory T cells traffic in the red pulp and patrol the MZ (Bajénoff et al., 2010; Schenkel et al., 2014), where RANKL is constitutively expressed by sessile MRCs (Jarjour et al., 2014; Katakai et al., 2008). However, naive T cells are primed in the follicular T cell zone requiring the migration of antigen-loaded DCs from the MZ to the periarteriolar lymphoid sheaths (PALSs).

Although the role of CD169[+] MZM in antigen transfer to B cells is well established (Martinez-Pomares and Gordon, 2012), few studies have addressed the role of these cells in cell-mediated immunity. Recent studies have shown that CD169[+] cells share the capacity for cross-presentation of cell-associated antigens to CD8 T cells with DCs (Asano et al., 2011; Bernhard et al., 2015) but can also transfer antigen to DC1 for cross-presentation (Backer et al., 2010). RANK deletion in lysozyme M (Lyz2)-expressing cells, which includes CD11c[+] macrophages but not DC1, resulted in the loss of CD169[+] MZMs but did not impair mCTL recall, implying that RANK function in CD11c[+] cells other than MZMs contributes to mCTL activation. DCs have previously been shown to be required for mCTL activation upon infection with bacterial and viral pathogens (Zammit et al., 2005). However, these studies utilized CD11c-DTR mice, in which both DCs and CD169[+] macrophages are lost (Bernhard et al., 2015; Probst et al., 2005). A more recent study showed that XCR1[+] DC1s are specifically required for mCTL activation in response to certain pathogens, including VSV (Alexandre et al., 2016). We showed that acquisition of VSV by DC1 in the spleen was significantly reduced after RANK deletion in CD11c[+] cells. Since DC1 numbers were not affected upon RANK deletion, this implies that VSV is transferred to DC1 via RANK-dependent MZMs. However, antigen targeted directly to DC1 still required RANK signaling for full activation of mCTLs, suggesting that RANK contributes to mCTL activation by DC1 independently of antigen transfer from MZMs. RANK signaling significantly increased antigen-specific CD8 T cell activation by DC *in vitro* in response to exogenous antigen, suggesting that RANKL/RANK signaling in the MZ may increase DC functionality and activation of mCTLs. In addition, RANKL can induce interleukin (IL)-12 and IL-15 expression in DCs (Josien et al., 1999), which are known to play important roles in the propagation of mCTL responses (Alexandre et al., 2016). The localized expression of RANKL in the MZ may, therefore, restrict the effects of RANK signaling to DCs in this specific niche and may even have a role in retention of RANK[+] DCs, thus favoring the activation of mCTLs and not the priming of naive T cells by migratory DCs.

In summary, we describe an important role for the RANKL/RANK signaling axis in the orchestration of macrophage and DC activation during the immune response to viral infection. RANK-dependent CD169[+] MZMs represent a niche for viral replication, and RANK signaling in DCs coordinately regulates mCTL recruitment and activation to propagate the immune response. These studies reveal new insights into how protective immunity to viral infection is triggered in the spleen MZ and the importance of RANK signaling in this specific niche.

EXPERIMENTAL PROCEDURES

Mice

Experiments were conducted in strict accordance with good animal practice according to French animal welfare bodies and the European Convention (EEC Directive 86/609) and were approved by the Direction Départmentale des Services Vétérinaires des Bouches du Rhônes. *Tnfrsf11a*$^{f/f}$ mice (Hanada et al., 2009) were crossed with Tg(*Itgax-Cre*) mice (Caton et al., 2007) or *Lyz2*$^{Cre/+}$ mice (Clausen et al., 1999) and subsequently backcrossed to wild-type C57Bl6/J mice for >12 generations. B6.Rag2^{tm1Fwa} (*Rag2*$^{-/-}$), B6.Rag2^{tm1Fwa}Tg(TcraTcrb)^{1100Mjb} (OT-I) mice were purchased from Taconic Biosciences. OT-I-EGFP mice have been previously described (Bajénoff et al., 2010). B6.SJL-Ptprca Pep3b/BoyJ (CD45.1[+]) mice were purchased from The Jackson Laboratory. CD45.1[+] mice were crossed with wild-type B6 mice (CD45.2[+]) to generate CD45.1[+]/CD45.2[+] mice for adoptive transfer experiments. *Rosa26*$^{lsl-tdRFP}$ mice and Tg(*Itgax-YFP*) mice have previously been described (Lindquist et al., 2004; Luche et al., 2007).

Virus and Bacteria

Recombinant VSV-OVA (Franck et al., 2012), recombinant Lm-OVA (Bajénoff et al., 2010), and VSV-EGFP (Iannacone et al., 2010) were kindly provided by L. Lefrancois, G. Lauvau, and J. Cunningham, respectively. VSV-EGFP was propagated at an MOI of 0.01 on BHK cells and purified as described previously (Franck et al., 2012; Iannacone et al., 2010). VSV titers from spleen were determined as described previously (Junt et al., 2007).

Preparation of OVA-Conjugated Monoclonal Antibodies

Purified rat immunoglobulin (Ig)G2a antibodies anti-CD169 (MOMA-1), anti-DEC205 (NLDC145), anti-DCIR2 (33D1), and isotype control (R7D4) were prepared as described previously (Backer et al., 2010).

Infections and Immunization

Mice were anesthetized using ketamine/xylazine before epicutaneous treatment with 62.5 mg 5% Aldara cream (IMQ; 3M Pharmaceuticals) and subcutaneous (s.c.) injection of 30 μL EndoGrade endotoxin-free chicken OVA (Hyglos) diluted in PBS (2 mg/mL). Primary infections with Lm-OVA (Bajénoff et al., 2010), VSV-OVA (Franck et al., 2012), or VSV-EGFP (Iannacone et al., 2010) were performed i.v.; Lm-OVA was grown to log phase in BD Bacto Brain Heart Infusion Broth (BHI; BD Biosciences ref. 237500), bacteria were washed in PBS, and 1 × 10^4 CFUs were injected per mouse. VSV-OVA and VSV-EGFP were injected at 10^5 or 10^6 PFUs per mouse. Secondary challenges were performed at least 2 months after IMQ/OVA immunization or primary Lm-OVA infection. OVA-conjugated monoclonal antibodies (Backer et al., 2010) were injected i.p. 1 hr after VSV infection.

Antibodies

Anti-CD3e (145-2C11), anti-CD11c (HL3), anti-CD4 (RM4-5), anti-CD8α (53-6.7), anti-CD11b (M1/70), anti-CD44 (IM7), anti-CD45.1 (A20), anti-CD45.2

(104), anti-CD45R (B220) (RA3-6B2), anti-CD40 (3/23), anti-CD254 (RANKL) (IK22-5), anti-CD265 (RANK) (R12-31), anti-CD169 (MOMA-A), anit-SIGN-R1 (ER-TR9), anti-B220 (RA3-6B2), anti-NK1.1 (PK136), anti-IFNγ (XMG1.2), anti-Granzyme B (FGB12), anti-CXCL9 (MIG-2F5.5), anti-Ly6C (AL-21), and anti-MHCII (M5/114) were purchased from BD Biosciences, eBioscience, BioLegend, and Life Technologies. H-2Kb-OVA$^{257-264}$ PE tetramers were purchased from Beckman Coulter Genomics.

Flow Cytometry

Single-cell suspensions from LNs or spleen were first incubated at 4°C for 10 min with the 2.4.G2 antibody to block Fc receptors and then stained with the indicated antibodies for 30 min at 4°C. Dead cells were gated out using SYTOX Blue dead cell stain (Life Technlogies, ref. S34857). For IFNγ and GrzB intracellular staining, spleen cells were stimulated with either 10 μM OVA$^{257-264}$/OVA$^{323-339}$ or ionomycin and phorbol 12-myristate 13-acetate (PMA) in RPMI 1640 medium containing 10% fetal calf serum (FCS) and GolgiStop (BD Biosciences) for 4 hr. After cell-surface staining, cells were fixed, permeabilized, and washed with BD Cytofix/Cytoperm or BD Perm/Wash buffer (BD Biosciences, ref. 554723/554722). Cells were then incubated with IFNγ and GrzB antibodies diluted in Perm/Wash buffer for 30 min at 4°C. Analysis was performed using BD FACSCanto or LSR-2 flow cytometers (BD Biosciences), and data analysis was conducted with the FlowJo cytometric analytical software (Tree Star).

Immunofluorescence Microscopy

Mice were killed 7 hr post-infection, and spleen was harvested and fixed for 4 hr in Antigenfix (DiaPath, MM France) at 4°C, washed in phosphate buffer (GIBCO, ref. 14190-094) for 1 hr, dehydrated in 30% sucrose overnight at 4°C, and embedded in optimum cutting temperature medium (OCT) freezing media (Tussue-Tek, ref. 4583). Spleen sections were blocked for 1 hr in 2% BSA before incubation with primary antibodies for at least 1 hr or overnight, depending on the antibodies. Stained spleen sections were then mounted in ProLong Gold antifade reagent with DAPI (Life Technologies, ref. P36931) and analyzed by confocal microscopy using the Zeiss LSM 780. Quantification of colored pixels was performed using ImageJ software. OT-I cell clustering was defined as a group of 3 or more proximate cells. The proportion of clustered cells was quantified according to the total number of OT-I GFP cells present on the whole section. The limits of the MZ region were determined with nuclear density and RANKL expression by MRCs lining the MZ. Staining was quantified only in the MZ by manual separation using ROI Manager tools in ImageJ. The same regions from each section were applied to all channels after specification of the limit threshold intensity for each channel. The mean gray value for all channels was measured using the ROI Manager tool.

Statistical Analysis

p values were calculated using either the Mann-Whitney test or the Fisher test, where appropriate, using GraphPad prism software. p values are indicated as: *$p < 0.05$; **$p < 0.01$; ***$p < 0.001$; and ****$p < 0.0001$.

SUPPLEMENTAL INFORMATION

Supplemental Information includes Supplemental Experimental Procedures and six figures and can be found with this article online at https://doi.org/10.1016/j.celrep.2017.10.111.

AUTHOR CONTRIBUTIONS

M.H. and T.L. designed the experiments. M.H. performed the experiments with help from O.R., C.V., L.C., and M. Bebien. J.M.P. provided *Tnfrsf11a$^{f/f}$* mice. S.A. provided VSV-OVA. J.M.M.d.H. provided OVA-coupled antibodies. M. Bajénoff made intellectual contributions. M.H. and T.L. wrote the manuscript.

ACKNOWLEDGMENTS

We thank Boris Reizis (Columbia University, USA) for Tg(*Itgax-Cre*) mice and Sean Whelan (Harvard Medical School, USA) and Matteo Iannacone (San Raffaele Scientific Institute, Italy) for VSV-EGFP. We also thank our colleagues; Marc Dalod, Karine Crozat, and Emilie Narni-Mancinelli for helpful advice and sharing reagents; and Michel Buferne and Noémie Clement for technical support. These studies were supported by grants to T.L. from L'Agence Nationale de la Recherche (ANR) (ANR-09-MIEN-029-01 and ANR-10-BLAN-1302-01); the European Research Council, FP7/2007–2013 grant agreement number 260753; and institutional funding from Inserm, CNRS, and Aix-Marseille-Université. Microscopy facilities are supported by ANR-10-INBS-04-01 (France-BioImaging).

Received: August 4, 2017
Revised: September 30, 2017
Accepted: October 28, 2017
Published: November 28, 2017

REFERENCES

Alexandre, Y.O., Ghilas, S., Sanchez, C., Le Bon, A., Crozat, K., and Dalod, M. (2016). XCR1+ dendritic cells promote memory CD8+ T cell recall upon secondary infections with *Listeria monocytogenes* or certain viruses. J. Exp. Med. *213*, 75–92.

Asano, K., Nabeyama, A., Miyake, Y., Qiu, C.H., Kurita, A., Tomura, M., Kanagawa, O., Fujii, S., and Tanaka, M. (2011). CD169-positive macrophages dominate antitumor immunity by crosspresenting dead cell-associated antigens. Immunity *34*, 85–95.

Backer, R., Schwandt, T., Greuter, M., Oosting, M., Jüngerkes, F., Tüting, T., Boon, L., O'Toole, T., Kraal, G., Limmer, A., and den Haan, J.M. (2010). Effective collaboration between marginal metallophilic macrophages and CD8+ dendritic cells in the generation of cytotoxic T cells. Proc. Natl. Acad. Sci. USA *107*, 216–221.

Bajénoff, M., Narni-Mancinelli, E., Brau, F., and Lauvau, G. (2010). Visualizing early splenic memory CD8+ T cells reactivation against intracellular bacteria in the mouse. PLoS ONE *5*, e11524.

Baratin, M., Foray, C., Demaria, O., Habbeddine, M., Pollet, E., Maurizio, J., Verthuy, C., Davanture, S., Azukizawa, H., Flores-Langarica, A., et al. (2015). Homeostatic NF-κB signaling in steady-state migratory dendritic cells regulates immune homeostasis and tolerance. Immunity *42*, 627–639.

Bernhard, C.A., Ried, C., Kochanek, S., and Brocker, T. (2015). CD169+ macrophages are sufficient for priming of CTLs with specificities left out by cross-priming dendritic cells. Proc. Natl. Acad. Sci. USA *112*, 5461–5466.

Brendolan, A., and Caamaño, J.H. (2012). Mesenchymal cell differentiation during lymph node organogenesis. Front. Immunol. *3*, 381.

Caton, M.L., Smith-Raska, M.R., and Reizis, B. (2007). Notch-RBP-J signaling controls the homeostasis of CD8- dendritic cells in the spleen. J. Exp. Med. *204*, 1653–1664.

Ciavarra, R.P., Buhrer, K., Van Rooijen, N., and Tedeschi, B. (1997). T cell priming against vesicular stomatitis virus analyzed in situ: red pulp macrophages, but neither marginal metallophilic nor marginal zone macrophages, are required for priming CD4+ and CD8+ T cells. J. Immunol. *158*, 1749–1755.

Clausen, B.E., Burkhardt, C., Reith, W., Renkawitz, R., and Förster, I. (1999). Conditional gene targeting in macrophages and granulocytes using LysMcre mice. Transgenic Res. *8*, 265–277.

Dalod, M., Chelbi, R., Malissen, B., and Lawrence, T. (2014). Dendritic cell maturation: functional specialization through signaling specificity and transcriptional programming. EMBO J. *33*, 1104–1116.

Edelson, B.T., Bradstreet, T.R., Hildner, K., Carrero, J.A., Frederick, K.E., Kc, W., Belizaire, R., Aoshi, T., Schreiber, R.D., Miller, M.J., et al. (2011). CD8α (+) dendritic cells are an obligate cellular entry point for productive infection by *Listeria monocytogenes*. Immunity *35*, 236–248.

Farrell, H.E., Davis-Poynter, N., Bruce, K., Lawler, C., Dolken, L., Mach, M., and Stevenson, P.G. (2015). Lymph node macrophages restrict murine cytomegalovirus dissemination. J. Virol. *89*, 7147–7158.

Franck, E., Bonneau, C., Jean, L., Henry, J.P., Lacoume, Y., Salvetti, A., Boyer, O., and Adriouch, S. (2012). Immunological tolerance to muscle autoantigens

involves peripheral deletion of autoreactive CD8+ T cells. PLoS ONE *7*, e36444.

Hammonds, J.E., Beeman, N., Ding, L., Takushi, S., Francis, A.C., Wang, J.J., Melikyan, G.B., and Spearman, P. (2017). Siglec-1 initiates formation of the virus-containing compartment and enhances macrophage-to-T cell transmission of HIV-1. PLoS Pathog. *13*, e1006181.

Hanada, R., Leibbrandt, A., Hanada, T., Kitaoka, S., Furuyashiki, T., Fujihara, H., Trichereau, J., Paolino, M., Qadri, F., Plehm, R., et al. (2009). Central control of fever and female body temperature by RANKL/RANK. Nature *462*, 505–509.

Honke, N., Shaabani, N., Cadeddu, G., Sorg, U.R., Zhang, D.E., Trilling, M., Klingel, K., Sauter, M., Kandolf, R., Gailus, N., et al. (2011). Enforced viral replication activates adaptive immunity and is essential for the control of a cytopathic virus. Nat. Immunol. *13*, 51–57.

Iannacone, M., Moseman, E.A., Tonti, E., Bosurgi, L., Junt, T., Henrickson, S.E., Whelan, S.P., Guidotti, L.G., and von Andrian, U.H. (2010). Subcapsular sinus macrophages prevent CNS invasion on peripheral infection with a neurotropic virus. Nature *465*, 1079–1083.

Jarjour, M., Jorquera, A., Mondor, I., Wienert, S., Narang, P., Coles, M.C., Klauschen, F., and Bajénoff, M. (2014). Fate mapping reveals origin and dynamics of lymph node follicular dendritic cells. J. Exp. Med. *211*, 1109–1122.

Josien, R., Wong, B.R., Li, H.L., Steinman, R.M., and Choi, Y. (1999). TRANCE, a TNF family member, is differentially expressed on T cell subsets and induces cytokine production in dendritic cells. J. Immunol. *162*, 2562–2568.

Josien, R., Li, H.L., Ingulli, E., Sarma, S., Wong, B.R., Vologodskaia, M., Steinman, R.M., and Choi, Y. (2000). TRANCE, a tumor necrosis factor family member, enhances the longevity and adjuvant properties of dendritic cells in vivo. J. Exp. Med. *191*, 495–502.

Junt, T., Tumanov, A.V., Harris, N., Heikenwalder, M., Zeller, N., Kuprash, D.V., Aguzzi, A., Ludewig, B., Nedospasov, S.A., and Zinkernagel, R.M. (2006). Expression of lymphotoxin beta governs immunity at two distinct levels. Eur. J. Immunol. *36*, 2061–2075.

Junt, T., Moseman, E.A., Iannacone, M., Massberg, S., Lang, P.A., Boes, M., Fink, K., Henrickson, S.E., Shayakhmetov, D.M., Di Paolo, N.C., et al. (2007). Subcapsular sinus macrophages in lymph nodes clear lymph-borne viruses and present them to antiviral B cells. Nature *450*, 110–114.

Katakai, T., Suto, H., Sugai, M., Gonda, H., Togawa, A., Suematsu, S., Ebisuno, Y., Katagiri, K., Kinashi, T., and Shimizu, A. (2008). Organizer-like reticular stromal cell layer common to adult secondary lymphoid organs. J. Immunol. *181*, 6189–6200.

Kim, S.K., Reed, D.S., Olson, S., Schnell, M.J., Rose, J.K., Morton, P.A., and Lefrançois, L. (1998). Generation of mucosal cytotoxic T cells against soluble protein by tissue-specific environmental and costimulatory signals. Proc. Natl. Acad. Sci. USA *95*, 10814–10819.

Kool, M., van Loo, G., Waelput, W., De Prijck, S., Muskens, F., Sze, M., van Praet, J., Branco-Madeira, F., Janssens, S., Reizis, B., et al. (2011). The ubiquitin-editing protein A20 prevents dendritic cell activation, recognition of apoptotic cells, and systemic autoimmunity. Immunity *35*, 82–96.

Li, J., Sarosi, I., Yan, X.Q., Morony, S., Capparelli, C., Tan, H.L., McCabe, S., Elliott, R., Scully, S., Van, G., et al. (2000). RANK is the intrinsic hematopoietic cell surface receptor that controls osteoclastogenesis and regulation of bone mass and calcium metabolism. Proc. Natl. Acad. Sci. USA *97*, 1566–1571.

Lindquist, R.L., Shakhar, G., Dudziak, D., Wardemann, H., Eisenreich, T., Dustin, M.L., and Nussenzweig, M.C. (2004). Visualizing dendritic cell networks in vivo. Nat. Immunol. *5*, 1243–1250.

Luche, H., Weber, O., Nageswara Rao, T., Blum, C., and Fehling, H.J. (2007). Faithful activation of an extra-bright red fluorescent protein in "knock-in" Cre-reporter mice ideally suited for lineage tracing studies. Eur. J. Immunol. *37*, 43–53.

Martinez-Pomares, L., and Gordon, S. (2012). CD169+ macrophages at the crossroads of antigen presentation. Trends Immunol. *33*, 66–70.

Moseman, E.A., Iannacone, M., Bosurgi, L., Tonti, E., Chevrier, N., Tumanov, A., Fu, Y.X., Hacohen, N., and von Andrian, U.H. (2012). B cell maintenance of subcapsular sinus macrophages protects against a fatal viral infection independent of adaptive immunity. Immunity *36*, 415–426.

Phan, T.G., Green, J.A., Gray, E.E., Xu, Y., and Cyster, J.G. (2009). Immune complex relay by subcapsular sinus macrophages and noncognate B cells drives antibody affinity maturation. Nat. Immunol. *10*, 786–793.

Probst, H.C., Tschannen, K., Odermatt, B., Schwendener, R., Zinkernagel, R.M., and Van Den Broek, M. (2005). Histological analysis of CD11c-DTR/GFP mice after in vivo depletion of dendritic cells. Clin. Exp. Immunol. *141*, 398–404.

Schenkel, J.M., Fraser, K.A., and Masopust, D. (2014). Cutting edge: resident memory CD8 T cells occupy frontline niches in secondary lymphoid organs. J. Immunol. *192*, 2961–2964.

Sewald, X., Ladinsky, M.S., Uchil, P.D., Beloor, J., Pi, R., Herrmann, C., Motamedi, N., Murooka, T.T., Brehm, M.A., Greiner, D.L., et al. (2015). Retroviruses use CD169-mediated trans-infection of permissive lymphocytes to establish infection. Science *350*, 563–567.

Steinman, R.M. (2008). Dendritic cells in vivo: a key target for a new vaccine science. Immunity *29*, 319–324.

Sung, J.H., Zhang, H., Moseman, E.A., Alvarez, D., Iannacone, M., Henrickson, S.E., de la Torre, J.C., Groom, J.R., Luster, A.D., and von Andrian, U.H. (2012). Chemokine guidance of central memory T cells is critical for antiviral recall responses in lymph nodes. Cell *150*, 1249–1263.

van der Fits, L., Mourits, S., Voerman, J.S., Kant, M., Boon, L., Laman, J.D., Cornelissen, F., Mus, A.M., Florencia, E., Prens, E.P., and Lubberts, E. (2009). Imiquimod-induced psoriasis-like skin inflammation in mice is mediated via the IL-23/IL-17 axis. J. Immunol. *182*, 5836–5845.

Weih, F., and Caamaño, J. (2003). Regulation of secondary lymphoid organ development by the nuclear factor-kappaB signal transduction pathway. Immunol. Rev. *195*, 91–105.

White, A., Carragher, D., Parnell, S., Msaki, A., Perkins, N., Lane, P., Jenkinson, E., Anderson, G., and Caamaño, J.H. (2007). Lymphotoxin a-dependent and -independent signals regulate stromal organizer cell homeostasis during lymph node organogenesis. Blood *110*, 1950–1959.

Zammit, D.J., Cauley, L.S., Pham, Q.M., and Lefrançois, L. (2005). Dendritic cells maximize the memory CD8 T cell response to infection. Immunity *22*, 561–570.

Zhang, N., and Bevan, M.J. (2011). CD8(+) T cells: foot soldiers of the immune system. Immunity *35*, 161–168.

Cell Reports, Volume *21*

Supplemental Information

Receptor Activator of NF-κB Orchestrates Activation of Antiviral Memory CD8 T Cells in the Spleen Marginal Zone

Mohamed Habbeddine, Christophe Verthuy, Olivia Rastoin, Lionel Chasson, Magali Bebien, Marc Bajenoff, Sahil Adriouch, Joke M.M. den Haan, Josef M. Penninger, and Toby Lawrence

Supplementary material

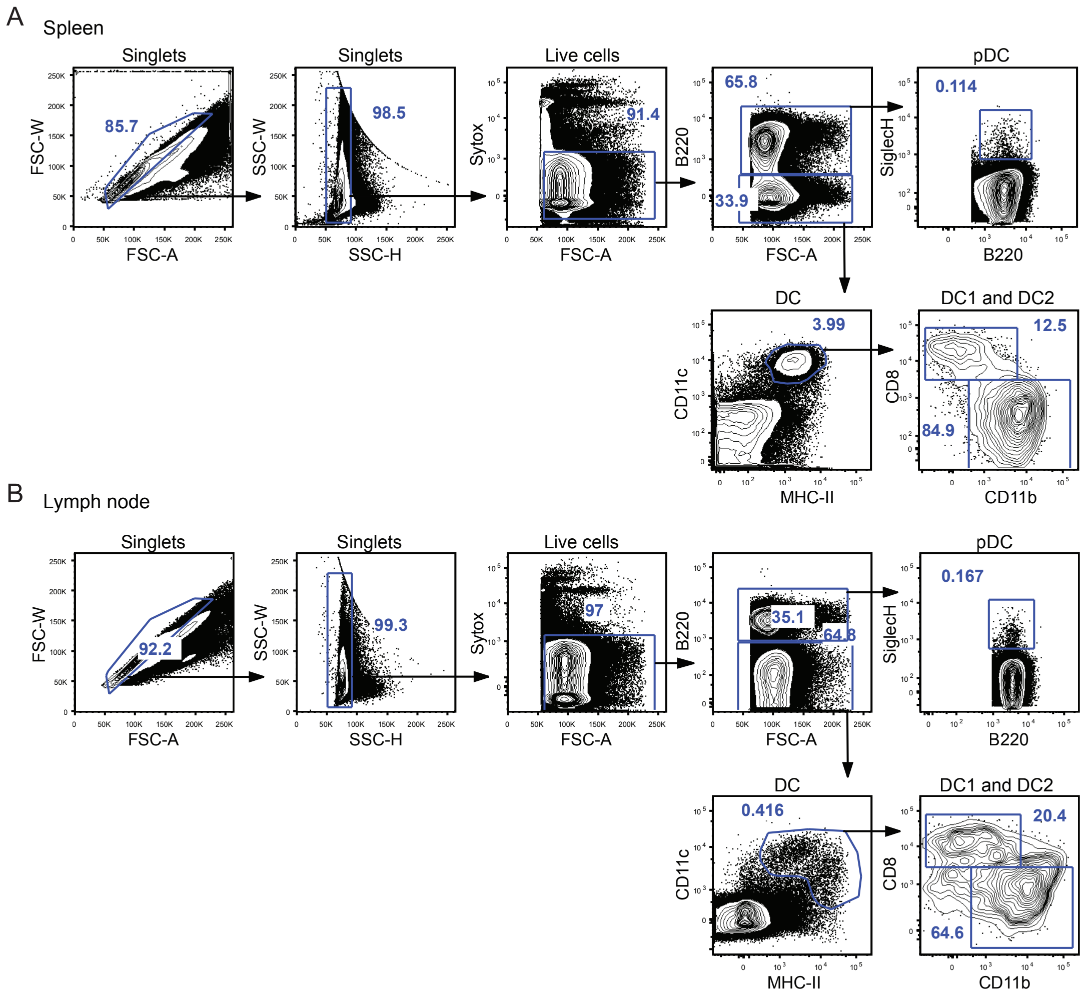

Figure S1. Detailed gating strategy for DC subsets, related to Figure 1. (A) gating strategy for DC1 (CD8α^{+}), DC2 (CD11b^{+}) and pDC (SiglecH^{+}) subsets in spleen (A) and lymph node (B).

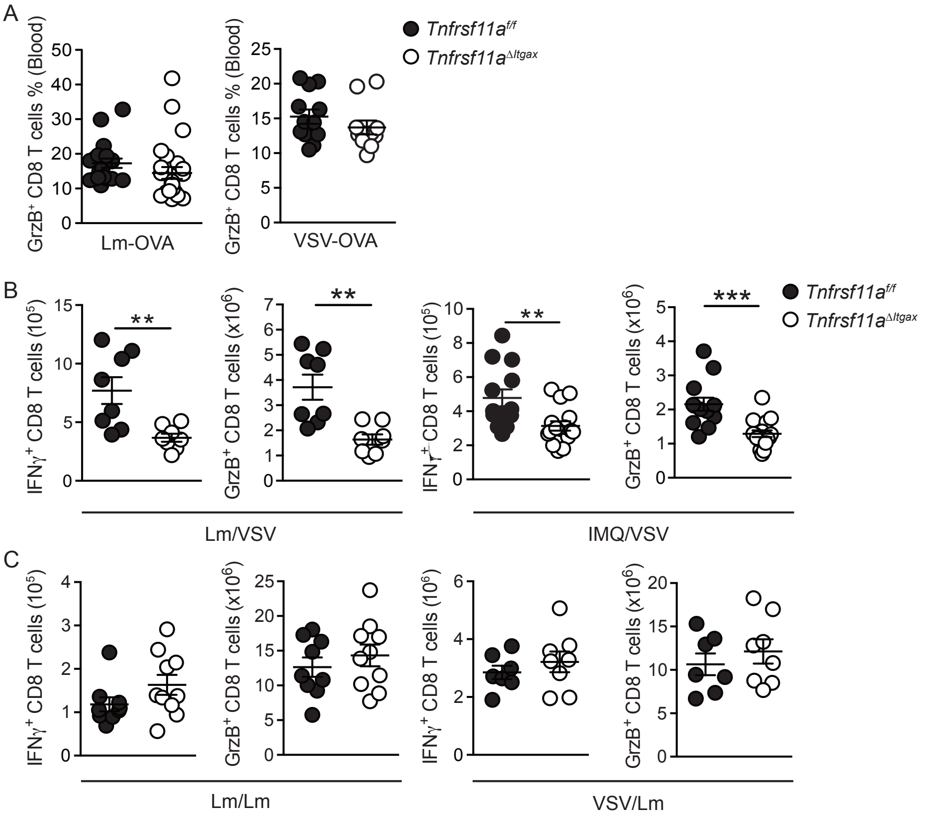

Figure S2. RANK expression by CD11c$^+$ cells regulates memory CD8 T cell activation in response to viral infection, related to Figure 2. (A) *Tnfrsf11a$^{f/f}$* and *Tnfrsf11a$^{\Delta Itgax}$* mice were infected i.v. with 1x10^4 Cfu Lm-OVA or 1x10^5 Pfu VSV-OVA; 1 week later blood was collected and granzyme B (GrzB) production by was measured in CD8$^+$ T cells by intracellular cytokine staining (ICS), after *ex vivo* stimulation with cognate peptide (SIINFEKL). (B-C) Cohorts of *Tnfrsf11a$^{f/f}$* and *Tnfrsf11a$^{\Delta Itgax}$* mice were immunized with Lm-OVA, IMQ/OVA or VSV-OVA; 2 months later mice were challenged with either VSV-OVA (B) or Lm-OVA (C), 5 days later, IFNγ and GrzB production were measured in spleen CD8$^+$ T cells by ICS, after *ex vivo* stimulation with SIINFEKL. Data are represented as mean ± s.e.m. and statistical analysis was performed with Mann–Whitney test; **p=<0.01, ***p=<0.005.

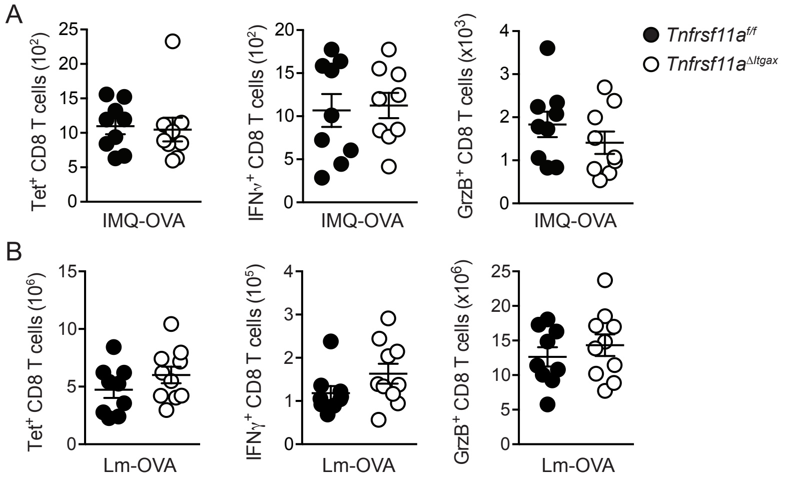

Figure S3. RANK in CD11c^{+} cells is not required for the development of pathogen-specific mCTL, related to Figure 2. (A-B) Cohorts of *Tnfrsf11a$^{f/f}$* and *Tnfrsf11a$^{\Delta Itgax}$* mice were immunized with IMQ/OVA (A) or Lm-OVA (B); 2 months later spleen cells were analysed by FACS for expansion of OVA-specific memory CD8 T cells (CD44^{+} Tet^{+}) and IFNγ or GrzB expressing cells upon activation with SIINFEKL. Data are represented as mean ± s.e.m.

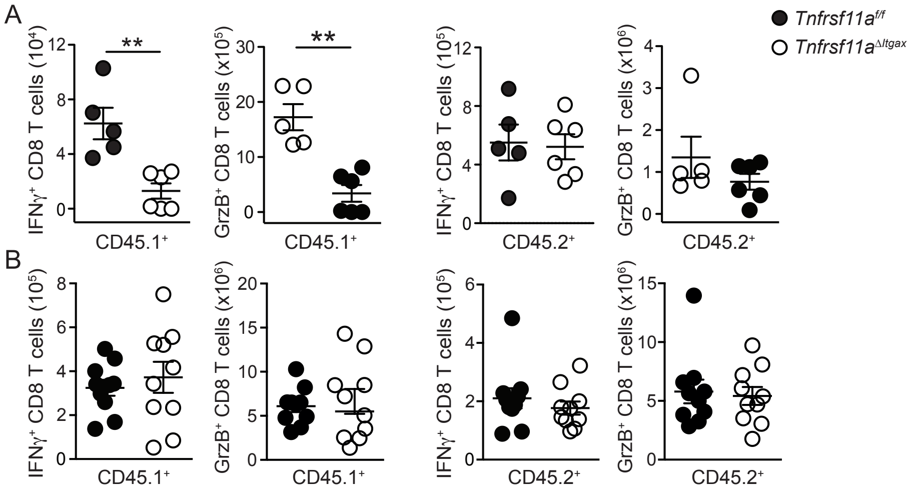

Figure S4. RANK in CD11c^{+} cells regulates activation of adoptively transferred memory CD8 T cells, related to Figure 2. (A-B) CD45.1^{+} mice were immunized with Lm-OVA, 3 weeks later CD4 and CD8 T cells were isolated from spleen and adoptively transferred to cohorts of naïve *Tnfrsf11a$^{f/f}$* and *Tnfrsf11a$^{\Delta Itgax}$* mice, a further 7 weeks after T cell transfer mice were infected with VSV-OVA (A) or Lm-OVA (B); IFNγ and GrzB expression in adoptively transferred mCTL (CD44^{+}, CD45.1^{+}) and endogenous naïve CD8 T cells (CD45.2^{+}) cells was measured 5 days after challenge in spleen, after stimulation with cognate peptide (SIINFEKL). Data are represented as mean ± s.e.m. and statistical analysis was performed with Mann–Whitney test; **p=<0.01.

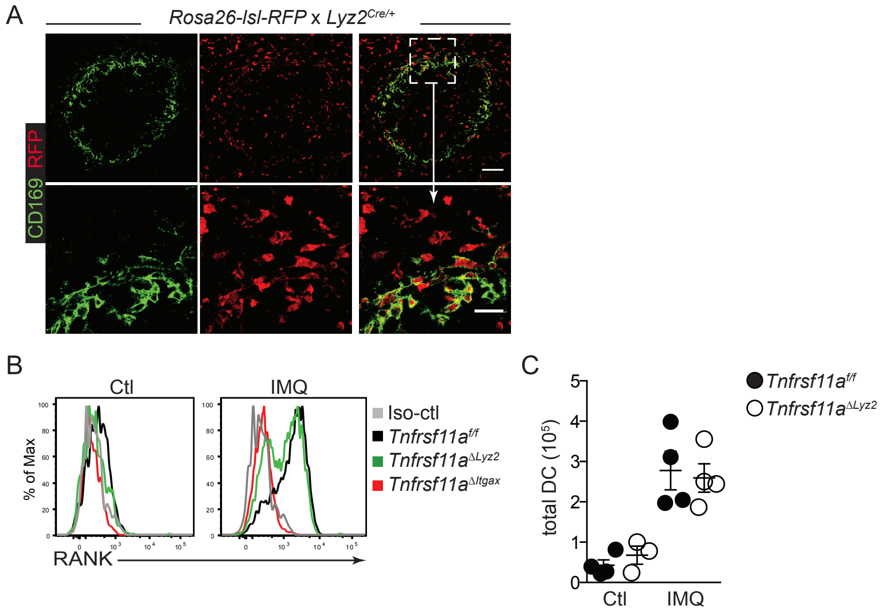

Figure S5. RANK expression in DC from *Tnfrsf11a*$^{\Delta Lyz2}$ mice, related to Figure 5. (A) Spleen sections from *Rosa26-LSL-tdRFP* x *Lyz2*$^{cre/+}$ mice showing Cre-recombinase activity in CD169^{+} cells; tdRFP (red) and CD169 (green). Scale bars; 50 μm in upper panels and 25 μm in lower panels. (B) RANK expression on DC (MHC-II^{hi} CD11c^{+}) was measured by flow cytometry in CLN from *Tnfrsf11a*$^{f/f}$, *Tnfrsf11a*$^{\Delta Lyz2}$ and *Tnfrsf11a*$^{\Delta Itgax}$ mice with and without IMQ treatment, representative FACs plots are shown. (B) Total number of DC were quantified. Representative data from at least 2 independent experiments are shown.

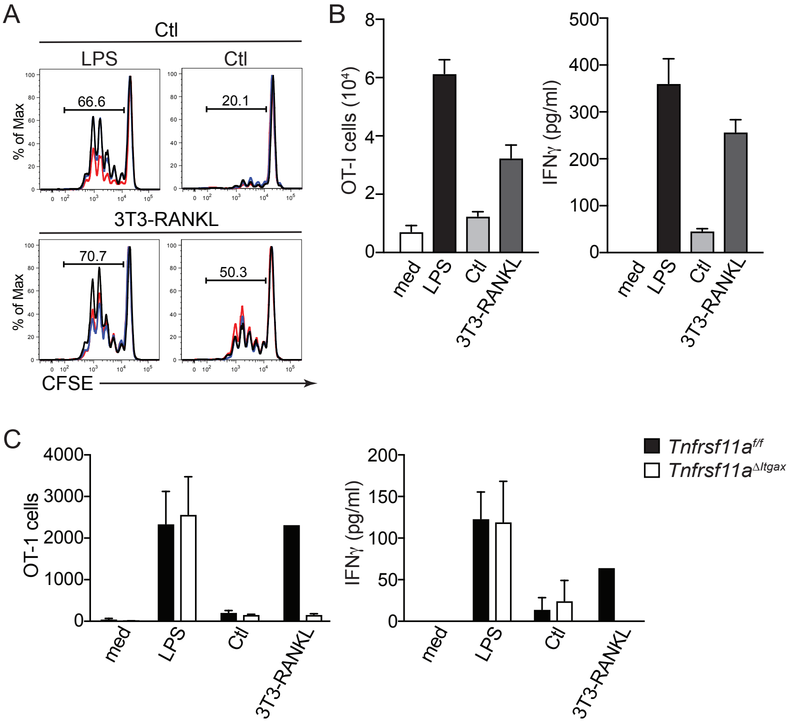

Figure S6. RANKL increases cross-presenting activity by DC *in vitro*, related to Figure 6. (A-B) Bone marrow-derived DC (BMDC) for wild-type mice (A-B), or *Tnfrsf11a*$^{f/f}$ and *Tnfrsf11a*$^{\Delta Itgax}$ mice (C), were co-cultured with mouse fibroblasts expressing RANKL (3T3-RANKL), control cells (Ctl) or 100ng/ml LPS, before loading with recombinant OVA and addition of CFSE labeled OVA-specific T cells (OT-1). After 3 days, CFSE dilution was analysed by flow cytometry and the number of proliferating OT-1 cells quantified. IFNγ production

by OT-1 cells was measured by ELISA. Data are represented as mean ± s.e.m. of cumulative data from 3 independent experiments.

Supplementary experimental procedures.

Generation of RANKL expressing stromal cells and cross-presentation assays.
3T3-RANKL cell line was generated as described by Sarrazin et al. (Sarrazin et al., 2009). Briefly, MSCV-based retroviral vectors were constructed by PCR cloning of cDNA for mouse RANKL under LTR control and by replacement of the IRES-driven GFP to generate RANKL-GFP. Viral supernatants was produced with ϕNXe cells (http://www.stanford.edu/group/nolan) and used to infect 3T3 cells by a double spin infection for 2 h in the presence of 8 mg/ml polybrene. Bone marrow-derived dendritic cells (BMDC) were generated according to the Steinman protocol (Inaba et al., 1992) . Briefly, cells were cultured with 20 ng/ml recombinant mouse GM-CSF (Peprotech) the medium was changed at day 2 and 4 with fresh medium supplemented with GM-CSF. BMDC were collected at day 6 and co-cultured overnight on top of 3T3-RANKL or Ctl cells at a ratio 45:1. Non-adherent DC were harvested and washed before incubation with recombinant OVA in culture medium for 1-2 hours. DC were then washed extensively with culture medium before addition of OT-1 T cells labelled with CFSE at a ratio 1:3 for 2.5 days, CFSE dilution as an index of T cell proliferation was analysed by flow cytometry.

Inaba, K., Inaba, M., Romani, N., Aya, H., Deguchi, M., Ikehara, S., Muramatsu, S., and Steinman, R.M. (1992). Generation of large numbers of dendritic cells from mouse bone marrow cultures supplemented with granulocyte/macrophage colony-stimulating factor. The Journal of experimental medicine *176*, 1693-1702.

Sarrazin, S., Mossadegh-Keller, N., Fukao, T., Aziz, A., Mourcin, F., Vanhille, L., Kelly Modis, L., Kastner, P., Chan, S., Duprez, E., *et al.* (2009). MafB restricts M-CSF-dependent myeloid commitment divisions of hematopoietic stem cells. Cell *138*, 300-313.
